# Graphene-Based Sensors for the Detection of Bioactive Compounds: A Review

**DOI:** 10.3390/ijms22073316

**Published:** 2021-03-24

**Authors:** Carlos Sainz-Urruela, Soledad Vera-López, María Paz San Andrés, Ana M. Díez-Pascual

**Affiliations:** 1 Universidad de Alcalá, Facultad de Ciencias, Departamento de Química Analítica, Química Física e Ingeniería Química, Ctra. Madrid‐Barcelona Km. 33.6, 28805 Alcalá de Henares, Madrid, España (Spain); carlos.sainz@uah.es (C.S.-U.); soledad.vera@uah.es (S.V.-L.); mpaz.sanandres@uah.es (M.P.S.); 2 Universidad de Alcalá, Instituto de Investigación Química Andrés M. del Río (IQAR), Ctra. Madrid‐Barcelona Km. 33.6, 28805 Alcalá de Henares, Madrid, España (Spain)

**Keywords:** bioactive compound, graphene, graphene oxide, melatonin, gallic acid, tannic acid, resveratrol, oleuropein, hydroxytyrosol, tocopherol, ascorbic acid, curcumin

## Abstract

Over the last years, different nanomaterials have been investigated to design highly selective and sensitive sensors, reaching nano/picomolar concentrations of biomolecules, which is crucial for medical sciences and the healthcare industry in order to assess physiological and metabolic parameters. The discovery of graphene (G) has unexpectedly impulsed research on developing cost-effective electrode materials owed to its unique physical and chemical properties, including high specific surface area, elevated carrier mobility, exceptional electrical and thermal conductivity, strong stiffness and strength combined with flexibility and optical transparency. G and its derivatives, including graphene oxide (GO) and reduced graphene oxide (rGO), are becoming an important class of nanomaterials in the area of optical and electrochemical sensors. The presence of oxygenated functional groups makes GO nanosheets amphiphilic, facilitating chemical functionalization. G-based nanomaterials can be easily combined with different types of inorganic nanoparticles, including metals and metal oxides, quantum dots, organic polymers, and biomolecules, to yield a wide range of nanocomposites with enhanced sensitivity for sensor applications. This review provides an overview of recent research on G-based nanocomposites for the detection of bioactive compounds, providing insights on the unique advantages offered by G and its derivatives. Their synthesis process, functionalization routes, and main properties are summarized, and the main challenges are also discussed. The antioxidants selected for this review are melatonin, gallic acid, tannic acid, resveratrol, oleuropein, hydroxytyrosol, tocopherol, ascorbic acid, and curcumin. They were chosen owed to their beneficial properties for human health, including antibiotic, antiviral, cardiovascular protector, anticancer, anti-inflammatory, cytoprotective, neuroprotective, antiageing, antidegenerative, and antiallergic capacity. The sensitivity and selectivity of G-based electrochemical and fluorescent sensors are also examined. Finally, the future outlook for the development of G-based sensors for this type of biocompounds is outlined.

## 1. Introduction

Carbon is an essential element on earth, which can be found in several structures in nature. The most usual and stable ones are diamond and graphite. Diamond has a rigid 3-D structure with sp^3^ carbon atoms arranged in a lattice, which is a variation of the face-centered cubic crystal structure. It has superlative physical qualities, most of which originate from the strong covalent bonding between its atoms. Graphite consists of a layered structure made of hexagonal rings of carbon atoms with sp2-hybridization. These layers are linked by Van der Waals forces that generate an exfoliating structure. Due to the weak forces between the graphite layers, it is possible to isolate one of these layers to obtain graphene (G), an atomically thick 2D sheet comprising sp2 carbon atoms arranged in a honeycomb structure. It has superior electronic, thermal, optical, and mechanical properties with values that surpass those obtained in any other materials [1,2]. For instance, it has exceptional thermal conductivity, in the range of 3000–5000 W·m^−1^·K^−1^ [3], superior to that of copper, which is around 400 W·m^−1^·K^−1^, very high electron mobility (25,000 cm^2^ V^−1^ s^−1^) [4], the highest electrical conductivity known at room temperature (6000 S cm^−1^) [5], a very large specific surface area (2640 m^2^ g^−1^) [6], and it is impermeable to gases. Further, it is a zero-gap semiconductor material, is electroactive and transparent, absorbing only 2.3% of the incident light. Moreover, G presents a Young’s modulus close to 1 TPa and an ultimate strength of 130 GPa, thus being the strongest material ever measured, stiffer than steel [7,8]. These unique properties make G an ideal candidate for a wide range of applications such as sensors, supercapacitors, fuel cells, photovoltaic devices, batteries, nanocomposites, flexible electronic devices, and so forth [9,10,11,12,13].

**Scheme 1:**
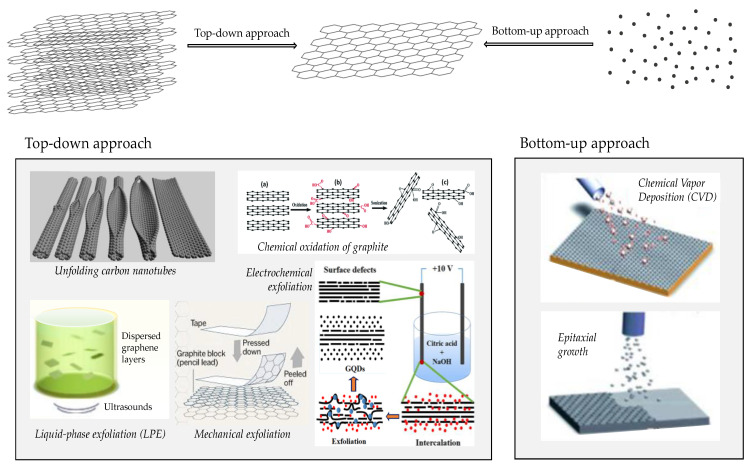
Representation of bottom-up and top-down approaches for G synthesis. GDQ: graphene quantum dots.

G is the starting point of other structures like fullerenes, nanotubes, or graphene quantum dots. It should be noticed that the term “graphene” used in the literature includes a broad range of graphene-like structures which differ in the preparation method and consequently in the chemical structure (usually the oxidation level), shape, size, and the number of layers. G synthesis is based on two general approaches, i.e., bottom-up and top-down approaches, as shown in Scheme 1. In the first approach, the starting material is graphite, and the aim is to exfoliate it via mechanical, liquid phase, or electrochemical exfoliations. Another approach in this group is to exfoliate graphite oxide to graphene oxide (GO), followed by chemical or thermal reduction. The bottom-up method is based on making graphene from molecular precursors building blocks by chemical vapor deposition (CVD) or epitaxial growth.

Mechanical exfoliation is the simplest technique, in which G is isolated by peeling it off from graphite flakes using tape. The main concern of this method is the low graphene quality and the limitation for large-scale production [2]. CVD is a scalable and cost-effective technique to produce high-quality graphene films, hence it is the most used to fabricate G at a large-scale, although it is difficult to attain a proper control of the G thickness. CVD uses the saturation of carbon by a high-tempered hydrocarbon gas on a transition metal substrate [14]. When the metal becomes cold, carbon solubility decreases, and carbon atoms precipitate, forming a G layer. Epitaxial growth enables control over the layer thickness by modifying the time and temperature throughout the process. However, it is one of the most expensive synthesis methods since a silicon carbide has to be heated between 1080 °C and 1320 °C to lead the growth of G; thus, it is not affordable at a large-scale. Moreover, the face of SiC used, *Si*-terminal or *C*-terminal, highly influences the thickness, mobility, and density of graphene [15]. Chemical oxidation of graphite is frequently used to obtain a graphene derivative, GO. Different oxidizing reagents can be used, like concentrated acids, such as KMnO_4_ or K_2_CrO_4_. The yield of graphene oxidation depends greatly upon the graphite source (flake size, crystallinity, purity, etc.) and can be tuned with other parameters such as oxidation time, acid ratios, mixing, washing, removal of non-oxidized material, etc. Subsequently, a reduction stage has to be applied to reduce the GO, although the resulting product differs significantly from raw G, and it is commonly named reduced graphene oxide (rGO) [16,17]. Another method to get a G layer is unfolding carbon nanotubes (CNTs) via chemical or plasma etching [18]. Other approaches include liquid-phase exfoliation (LPE), which uses specific organic molecules, or electrochemical exfoliation, which relies on the penetration of graphite by ions from the electrochemical solution using a potential [19]. Depending on the synthesis method, graphene features are different, as well as the yield and the costs [20,21]. In addition, it should be noted that due to graphene forces, it tends to fold or join with other G layers, leading to an agglomerated nanomaterial. For this reason, surfactants are usually required to attain stable G monolayers in solution [22,23].

**Scheme 2:**
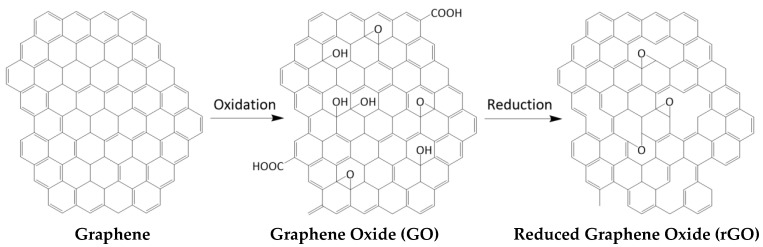
Molecular structures of Graphene, Graphene Oxide, and Reduced Graphene Oxide.

Some researchers have discovered that GO and rGO have better properties for some applications than graphene. GO is a modified form of G that comprises carboxylic groups on the edges and epoxy and hydroxyl groups on the layer plane (Scheme 2). It can be obtained via chemical oxidation graphite [24], as we mentioned before, via oxidation of G using the Hummers’ method [17,25] or by electrochemical exfoliation of graphite oxide GO [26,27], leading to a larger surface, which allows keeping compounds in the interlayer space. This property is especially useful for biomedical applications.

On the other hand, rGO is obtained via the thermal treatment of GO to remove functional groups [24] or by chemical reduction of GO (Scheme 2). Reduction of the epoxide groups of the GO can be conducted by using hydrazine or sodium borohydride as reduction agents, but the dehydroxylation and decarboxylation need heat treatment (as an endothermic reaction). However, these components are corrosive, combustible, and highly toxic, which may be dangerous for personnel health and the environment. Hence, eco-friendly, natural reducing agents are sought, such as aminoacids (i.e., ascorbic acid) or plant extracts (i.e., Ginkgo biloba leaves).

GO is removed more easily, both kinetically and thermodynamically; rGO has improved the electric properties compared to GO, due to the reduced amount of functional groups, although its retention capability is lower [17]. The functional groups of both G and GO enable them to be functionalized and to interact with other materials [28].

**Figure 1:**
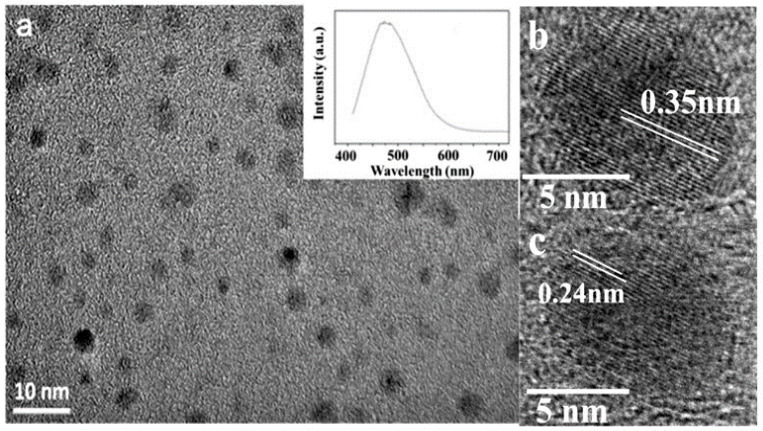
(**a**) TEM images of graphene quantum dots (GQDs) of different sizes and the photoluminescence spectrum excited at 400 nm. (**b**,**c**) High-resolution transmission electron microscopy (HRTEM) images of GQDs. Taken from Wu et al. [32].

On the other hand, graphene quantum dots (GQDs) have recently emerged amongst the family of carbon nanomaterials. They are 0D graphene sheets, with circular planar geometry and very small size, about 3–20 nm (Figure 1). Due to the confinement of the excitons and the quantum effect, GQDs exhibit many excellent properties such as chemical inertness, photobleaching resistance, and stable luminescence. Thus, photoluminescence can be induced in graphene material when the size, structure, and surface property are controlled properly as for traditional QDs [29]. Fluorescent GQDs have great potential for application in bioimaging, diagnosis, and drug delivery and can be used in Föster resonance energy transfer (FRET) optical sensors as donors [30]. Further, GQDs combined with conventional plasmonic material such as noble metals can significantly improve the absorption (and interaction) with visible and IR light, which is only 2.3% for bare G. Besides, GQDs exhibit low toxicity, high conductivity, and good biocompatibility, which combined with their cost-effectiveness make them suitable and efficient in both optical and electrochemical sensing applications [31,32].

**Scheme 3:**
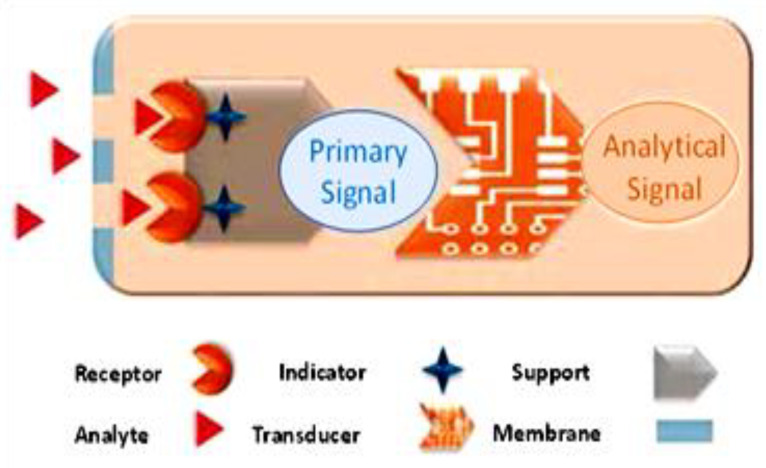
Basic representation of a chemical sensor.

A chemical sensor is innately defined as a device that responds to changes in the local chemical environment via an electrical, electrochemical, and/or optical signal. It transforms the chemical information of a sample (concentration of one or more of its components) into an analytically useful signal. The main parts of a chemical sensor shown in Scheme 3.

(1) A receptor zone has the role of transforming the chemical information into a form of primary energy or signal. It is the chemical part of the sensor and generally contains a reagent immobilized on a solid support. If the receptor is not capable of generating the primary signal by itself, it also contains an indicator. If the reagent is biological in nature and is called a biosensor, if it is synthetic, the term chemoreceptor is often used. It should be pointed out that, traditionally, the term biosensor also applies to chemoreceptors that act in a similar way to affinity bioreactants; that is, they have both the ability to recognize the analyte (molecular recognition) as well as to chemically interact with it, as is the case with Molecularly Imprinted Polymers (MIPs), or with non-natural reagents but of biological origin (aptamers or plastic antibodies). The selectivity, sensitivity, and precision of the sensor depend essentially on this component.

(2) A transducer zone, which transforms the primary signal into the final analytical signal, generally an electric current or a potential difference directly or inversely proportional to the analyte concentration. It is the most technical part of the sensor since, together with the transducer itself, it contains the necessary components for the transformation and treatment of the signals. Although there are different types of primary signals (mass, conductivity, magnetic, etc.), the most common transducers are electrochemical and optical. In the latter, the transducer is called a detector.

(3) A membrane to separate the whole sensor from the outside.

For a chemical sensor to be useful, its response must be reproducible, stable, sensitive, and selective. Over the last years, a change in sensor technology towards more sensitive elements, complex architecture, and size reduction has arisen due to the emergence of nanotechnology, that is, the science that deals with the manipulation of matter on the scale of atoms and molecules. Nanomaterials such as graphene [33], carbon nanotubes [34], metal oxide nanoparticles [35], and their polymeric composites [36] have been used to develop sensors to detect a variety of analytes.

Bioactive ingredients of natural products can protect the human body from harm, as well as prevent and treat disease. Screening bioactive compounds from natural products is attracting particular attention in a broad range of applications, including pharmacology, cosmetics, the food industry, biomedicine, and so forth. It is a very promising research area in full development, which has resulted in many studies devoted to diversify the resources of bioactive compounds and improve their synthesis or recovery pathways. However, despite all this significant research in various fields, the definition of bioactive compounds remains ambiguous and unclear. The term “bioactive” arises from bio- from the Greek (βίο-) “bios”, which means life, and active from the Latin “activus”, which means: dynamic or full of energy. In a strictly scientific sense, the term “bioactive” is an alternative term for “biologically active” [37]. Thus, a bioactive compound is just a substance that has biological activity [38]. These compounds contain chemicals that are found in small amounts in plants or certain foods such as fruits, vegetables, nuts, oils, and whole grains; further, they typically have an associated beneficial effect on health. It should be noted that in addition to natural bioactive substances [39], there are also synthetic bioactive molecules [40].

The benefits of G-based sensors can be summarized as follows: the high specific surface area and atomic thickness of G layers enable direct contact between the carbon atoms and the analytes; as a result, graphene-based sensors have better sensitivity compared to silicon [41]. In addition, the flexibility combined with the high optical transparency and electrical conductivity of G enables the acquisition of high-quality signals without motion artifacts or visual disturbances. Furthermore, a high signal-to-noise ratio can be achieved in electrophysiological signals due to the efficient signal transmission attained by the high electrical conductivity [42]. Besides, the large specific surface area, the feasibility of functionalization, and a high electron transfer rate enable receptors such as enzymes, antibodies, and DNA to be effectively immobilized on a G surface [43]. Consequently, many sensors based on G nanocomposites and their derivatives have been reported, including wearable sensors and implantable devices, that enable performing quick, real-time analysis [44] and are also applied in biomedicine for diagnosis, prognosis, prediction, and treatment methods. It is a novel technology, increasingly employed, in which it is completely essential to work at the nanoscale.

Although a number of reviews dealing with G-based sensors for the detection of biomolecules have been published, most of them are too general and deal only with electrochemical sensors. Further, they are devoted mainly to target biological molecules such as DNA, urea, glucose, and so forth. In this review, an attempt is made to show the state-of-art in the synthesis and performance of G-based fluorescent and electrochemical sensors for the specific detection of bioactive compounds, providing insights into the unique advantages offered by graphene and its derivatives. Their synthesis process (including functionalization routes), novel structures, and main properties are summarized, and the main challenges are also discussed. The reported works try to improve the conventional procedures via the use of cheaper, faster, more effective, or more eco-friendly methods.

## 2. Bioactive Compounds: Properties and Applications

Bioactive compounds are substances that typically occur in small quantities in food and can be beneficial for health. They are intensively studied to evaluate their effects, including antioxidant, antiallergic, antimicrobial, antithrombotic, antiatherogenic, hypoglycaemic, anti-inflammatory, antitumor, cytostatic, immunosuppressive properties, and hepatoprotective activities [45]. Unlike essential macro- and micronutrients, they are not essential for life, and the body can function properly without them.

Among the most common bioactive compounds found in our diets are polyphenols, which are well known for their antioxidant properties [46]. They inhibit or interfere with the process of free radical formation, preventing the oxidation of cells. Dietary antioxidants can protect the body from oxidative damage that may result over time in many pathologies such as cancer or cardiovascular disease. It is believed that polyphenols may exert a cardio protective effect via several paths. They may enhance the functioning of the inner lining of blood vessels, hinder platelet aggregation (preventing blood accumulations in the arteries), and positively influence blood lipids and insulin sensitivity [47]. Typical dietary antioxidants are vitamin C, vitamin E, and carotenoids, and most of them come from vegetables like olive, rice, broccoli, eggplant, ginger, onion, citrus, coffee, tomatoes, etc.

The antioxidant potential of phenolic compounds depends on the number and arrangement of the hydroxyl groups [48]. Phenolic antioxidants can give hydrogen atoms to lipid radicals and produce lipid derivatives and antioxidant radicals (Equation (1)), which are more stable and less readily available to promote autoxidation. The antioxidant free radical could then interfere with the chain-propagation reactions (Equations (2) and (3)).

(1) R∗/RO∗/ROO∗ + AH → A∗ + RH/ROH/ROOH

(2) RO∗/ROO∗ + A∗ → ROA/ROOA

(3) ROO∗ + RH → ROOH + R∗

As the hydrogen bond energy in a free radical scavenger (FRS) decreases, the hydrogen transfer to the free radical is energetically more favorable, hence faster. Any compound that has a reduction potential lower than that of a free radical (or oxidized species) is able to donate its hydrogen atom to that of the free radical unless the reaction is kinetically unfeasible. For instance, FRS including α-tocopherol (E°′  =  500 mV) that have a reduction potential lower than that of peroxyl radicals (E°′  =  1000 mV) is able to donate their hydrogen to the peroxyl radical to form a hydroperoxide [49]. The phenoxyl radical is then stabilized by the delocalization of its unpaired electron around the aromatic ring.

The influence of the antioxidant concentration on the autoxidation rate is conditioned by many factors such as the antioxidant structure, oxidation conditions, and nature of the sample being oxidized [50]. Frequently phenolic antioxidants lose their activity at high concentrations and are more effective in extending the induction period when added to any oil that has not deteriorated to any great extent. Thus, to attain the best protection against oxidation, antioxidants should be added to foodstuffs as early as possible during processing and storage. Nonetheless, the effect of polyphenols on humans is still not clear, and many studies claim that the beneficial effect arises from the polyphenol-rich foods rather than the isolated polyphenols. Up to date, the European Food Safety Authority (EFSA), which evaluates health statements made on food products, has rejected all claims on polyphenols except for olive oil, which contains hydroxytyrosol, and contributes to the protection of blood lipids from oxidative stress.

**Scheme 4:**
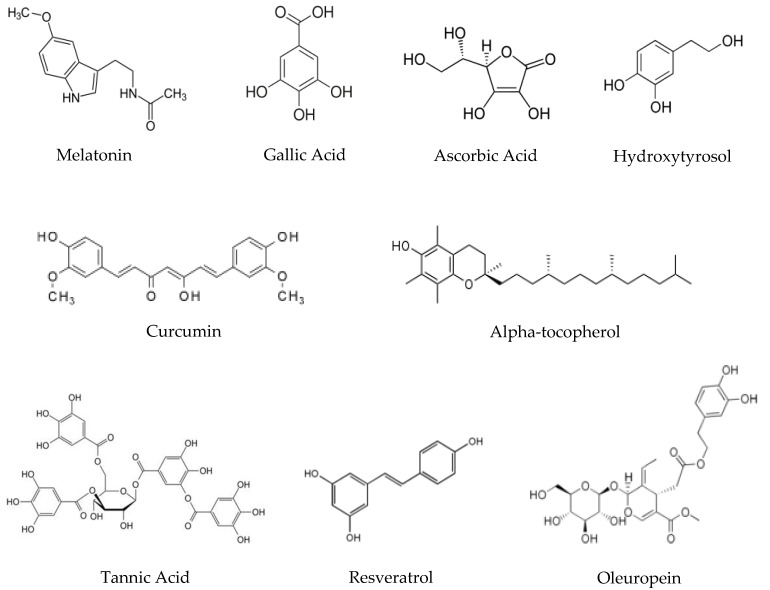
Chemical structure of the selected antioxidants.

Among natural phenolic antioxidants are phenolic acids, flavonoids, coumarins, stilbenes, hydrolyzable and condensed tannins, lignans, and lignins, and contain one or more hydroxyl groups attached directly to an aromatic ring. The antioxidants selected for this review were red wine polyphenolic compounds, including gallic acid, tannic acid, resveratrol, oleuropein, hydroxytyrosol, tocopherol, and ascorbic acid (the last two found in lower amounts). Further, melatonin, an indole amine also present in wine that shows a wide range of anticancer activities, and curcumin, a polyphenol with multiple health benefits, has also been addressed. All these antioxidants are either autofluorescent or can be derivatized to form a fluorescent compound. Their chemical structure is displayed in Scheme 4.

### 2.1. Melatonin

Melatonin, *N*-acetyl-5-methoxytryptamine (MLT), is a pleiotropic neurotransmitter with cellular and physiological actions, widely distributed in nature [51]. MLT is a hormone released primarily by the pineal gland that regulates the sleep-wake cycle. As a dietary supplement, it often is used in the short-term treatment of insomnia. This molecule is involved in circadian rhythms, hematopoiesis, angiogenesis, metastasis, sexual behavior, hypertension, oxidative stress, metabolic syndrome, and immune function. Furthermore, one of its most important activities is that it is a potent free radical detoxifier and regulator of redox-active enzymes [52]. The hydrophilic and lipophilic character of MLT has allowed it to cross the blood-brain barrier and to enter into cells, which have given this hormone the capacity of developing all the mentioned implications in animals [53]. Moreover, MLT improves the antioxidant reaction stimulating other antioxidative molecules such as glutathione peroxidase, glutathione reductase, SOD, and catalase [54]. Recent studies have shown that MLT deficiency causes a gradual acceleration of aging [55]. Furthermore, it presents immunomodulatory, thermoregulatory, and antitumor properties and appears to be an excellent candidate for the prevention and treatment of various cancers such as skin, breast, prostate, and colon [56].

### 2.2. Gallic Acid

Gallic acid (GA, 3,4,5-trihydroxybenzoic acid) belongs to the group of hydrolyzable tannins, and to the subclass, gallotannins. It is considered one of the strongest natural antioxidants. Gallic acid name is derived from oak galls, which are historically used to get tannins. It is distributed in different families of the vegetable kingdom, such Anacardiaceae, Myrtaceae, and Fabaceae, as well as the fungi genus such *Aspergillus*, *Penicillium*, and *Termitomyces* [57,58].

Its synthesis can be performed via three possible routes. One starts with the conversion of phenylalanine in caffeic acid, later in 3,4,5-trihydroxycinnamic acid, and then in gallic acid. Another starts from the artificial production of 3,4,5-trihydroxycinnamic acid, and its side chain comes from the formation of protocatechuic acid, which is derived from caffeic acid [57]. The third route uses 3-dehydroshikimate, in which the action of the enzyme shikimate dehydrogenase produces 3,5-didehydroshikimate. This compound tautomerizes to form a redox gallic acid equivalent, which is converted into GA due to its spontaneous aromatization [59,60]. Several beneficial effects are reported for gallic acid, including antioxidant, anti-inflammatory, and antineoplastic properties. Further, this compound has been reported to have therapeutic activities in gastrointestinal, neuropsychological, metabolic, and cardiovascular disorders.

### 2.3. Tannic Acid

Tannic acid (TA, penta-m-digallolyl glucose) is one of the main tannins in plants. It has multiple applications in medicine due to its antioxidant, antimutagenic, antiallergenic, and anticarcinogenic capacity [61]. Further, it is widely used in the wine and food industries. However, in Europe is not considered a food additive due to its toxicity in large amounts in animals [62]. Conversely, TA with magnesium was used as a treatment for many toxic substances in the early twentieth century. During that time, tannic acid was also applied to treat burn injuries. Nowadays, it is used in pharmacology to develop numerous applications. 

It has bactericide action since it reacts with proteins irreversibly, thus complexing within bacterial membranes, neutralizing their activity [63], and has effective anticaries properties. Its antiviral effectiveness is also well documented. Besides, it is used for the treatment of intestinal problems, owed to its complexation ability with other molecules and antioxidant behavior, and has been proven to be effective against ulcers by functioning as a protective coating of the gastrointestinal tract [64]. TA is extracted from several plant species like *Caesalpinia spinosa*, *Rhus semialata*, *Quercus infectoria*, and *Rhus coriaria*.

### 2.4. Resveratrol

Resveratrol (3,5,4′-trihydroxystilbene, RSV), a natural member of the stilbene family, has been reported to have numerous health benefits. It is present in several plants and fruits, such as peanuts, mulberries, blueberries, and, above all, in grapes, specifically in their skin, seeds, and woody parts [65,66,67]. Drinking red wine, unlike white wine where these rich parts in resveratrol are removed, could be responsible for health-promoting properties [68,69]. It is a phytoalexin, that is, an antimicrobial and antioxidative substance synthesized by a plant in response to environmental stresses, and it is used for the treatment of various diseases, including dermatitis, gonorrhea, fever, hyperlipidemia, arteriosclerosis, and inflammation. Furthermore, the antiproliferative and proapoptotic effects of resveratrol in tumor cell lines have recently been documented in vitro [70]. For all these reasons, RSV is one of the most studied antioxidants.

Its structure was not characterized until 1940 when Taraoka isolated it from *Veratrum grandiflorum* roots [71]. However, its properties have been used in medicinal preparations for 2000 years [72]. RSV has two isomers, one *trans* and another *cis*, being the first more stable and, thus, more abundant in nature. The RSV synthesis starts with phenylalanine, which after being modified by three enzymes, it is turned to cumaril-CoA. This product interacts with three molecules of malonil-CoA thanks to a stilbene synthetase, the resveratrol synthetase, generating the *trans*-resveratrol molecule [73]. A remarkable fact is that the synthesis of resveratrol decreases with the grape maturation, because of the lower genetic expression induction.

Some of the species with more quantity of resveratrol, besides the grapes, are *Polygonum cuspidatum* [74], *Bauhinia racemose* [75], *Veratrum grandiflorum* [76], *Veratrum formosanum* [77], *Pterolobium hexapetallum* [78], eucalyptus [79,80], and fir species [81]. RSV protects them from infections, UV radiation, chemical substances or stress [73,82,83].

### 2.5. Hydroxytyrosol and Oleuropein

Hydroxytyrosol (2-(3,4-dihydroxyphenyl)ethanol, HT) is a diphenolic compound naturally occurring in olives and olive oil, frequently occurring in the Mediterranean diet, with proven benefits for health [84,85,86]. It was first recognized by Stoll et al. as a component of echinacoside, a phenolic glycoside showing antibiotic activity against *Staphylococcus aureus* extracted from the roots of *Echinacea angustifolia* [85,87]. HT is considered the most powerful antioxidant after gallic acid. It has been most studied and used in food, cosmetic, and pharmaceutical industries [88] after being demonstrated that HT produces a wide range of biological properties besides antioxidant such as hepatoprotective [89], cytoprotective [90], neuroprotective [91], cardioprotective [92], anti-inflammatory [93], antiviral [94], anticancer [85], and anti-obesity effect [95]. Some studies affirm that the composition of olive leaves extracts rich in several phenols and flavonoids and that it varies with the harvesting season, the leaves maturity, storage conditions, and extraction method [96].

The antioxidant activity of HT in vivo is directly linked to its high bioavailability, and several works have reported a high degree of absorption, fundamental to exert its metabolic and pharmacokinetics properties [97]. HT behaves as an antioxidant acting as a free radical scavenger and radical chain breaking as well as a metal chelator. With its catecholic structure, it is able to scavenge the peroxyl radicals and break peroxidative chain reactions, leading to very stable resonance structures. On the other hand, the protection against the genotoxic action of reactive oxygen species (ROS) is one of the mechanisms explaining the anticancer effects of HT [98]. In addition, it may also act via the modulation of pro- and anti-oncogenic signaling pathways, resulting in cell apoptosis and growth restriction of tumor cells, which may be mediated by their capability to induce the accumulation of hydrogen peroxide in the culture medium [99]. A decrease in ROS production, derived by iron or copper-induced oxidation of low-density lipoproteins, has also been found, suggesting a chelating action on such metals.

HT can be obtained in several ways differing in the starting molecule and the synthetic route. The first synthesis path was reported by Schöpf et al. in 1949. It started with 2-(3,4-dimethoxyphenyl)ethanol that was demethylated by 48% HBr, leading to the corresponding bromide, which was converted into the triacetoxy derivative and subsequently hydrolyzed by methanolic NH_3_ to give hydroxytyrosol [85,100].

One of the derivates of hydroxytyrosol is oleuropein, which presents very similar properties: antioxidant, antimicrobial, antitumoral, and cardioprotective. Oleuropein (2-(3,4-dihydroxyphenyl)ethyl(2S,3E,4S)-3-ethylidene-2-(β-d-glucopyranosyloxy)-5-methoxycarbonyl)-3,4-dihydro-2*H*-pyran-4-yl)acetate) arises from the union of hydroxytyrosol and an oleosidic skeleton. It is a glycosylated seco-iridoid that can be found in green olive skin, flesh, seeds, and leaves [101]. During its maturation, the β-glucosidases present inside transforms the oleuropein to extra virgin olive oil, a form in which it is protected from natural oxidation. Similar to HT, it shows a beneficial effect on the reduction in cholesterol and the decrease in coronary heart diseases [102]. Besides, its consumption, mostly in the Mediterranean diet, reduces the risk of breast, prostate, and colon cancer, owed to its potential antitumoral activity [103,104,105,106,107,108].

### 2.6. Tocopherol

Tocopherol (TCP or TOH) is the name of a class of organic chemical compounds having some vitamin E activity. It is one of the 13 essential vitamins in humans that is not produced by the organism. Four forms of tocopherol with vitamin E activity and different degrees of methylation have been described: alpha-, beta-, gamma- and delta-tocopherol. α-tocopherol is a component integrated into the European diet because it is present in olive and sunflower oils [109]. Conversely, γ-tocopherol is more common in the American diet, where soybean and corn oil are intaken [109,110]. Fortunately for Europeans, α-tocopherol is the most absorbed form of vitamin E in humans [111].

One of the main functions of TCP is the capacity of donating an H atom to free radicals that could generate damage [112], resulting in a less reactive tocopheryl radical. Besides, TCP has anti-inflammatory, antiageing, anticancer, and cardioprotective properties, and its aids in preventing macular degeneration, platelet aggregation, and Alzheimer’s disease. It is widely used in moisturizers, creams, and as a food additive.

Unlike vitamins A and D, α-tocopherol (which is also a fat-soluble vitamin) does not accumulate to “toxic” levels in the liver or extrahepatic tissues; however, an excess of vitamin E arises few problems if the doses overcome 400 mg/day. It can be a topic allergic, normally seen in cosmetic products, although the incidence is quite low. It could generate a drug interaction such as tamoxifen, an anti-breast cancer drug, or cyclosporine A, an immune-suppressant drug, or aspirin and warfarin, potentiating an anti-blood-clotting action [113]. For some of these interactions, its co-administration together with a chemotherapy drug is completely forbidden.

The natural biosynthesis of TCP in plants comes from the reaction of homogentisate (HGA) with homogentisate phytyltransferase (HPT), giving a molecule that reacts with tocopherol cyclase (TC) to get δ-tocopherol or with methyltransferase (MT) and then tocopherol cyclase (TC) to get γ-tocopherol. Subsequently, if these two tocopherols react with γ-tocopherol methyltransferase (γ-TMT), β-tocopherol and α-tocopherol are obtained, respectively [114].

α-tocopherol can be extracted and purified from seed oils. In addition, γ-tocopherol can be extracted, purified, and methylated to produce α-tocopherol. It has been found that rats can convert γ-tocopherol to α-tocopherol, methylating it in their tissues. On the other hand, α-tocopherol can be obtained synthetically, although with only 50% of the effectiveness of natural α-tocopherol.

### 2.7. Ascorbic Acid

Ascorbic acid ((*R*)-3,4-dihydroxy-5-((*S*)-1,2-dihydroxyethyl)furano-2(5*H*)-one, AA) is an organic acid with antioxidant properties. The S enantiomer (l-ascorbic acid) has vitamin C activity. AA is very common in the diet, and it is usually sold as a dietary supplement. It prevents illnesses such as scurvy [115], which gave it the name of ascorbic acid or ascorbate. Vitamin C is an essential nutrient in humans that acts as a cofactor of several enzymes, promotes carnitine and collagen synthesis, and plays a crucial role in cell division and growth regulation, in the maintenance of the immune system, in the reparation of skin and tissues and in the production of neurotransmitters. Further, it presents antiageing, anticancer, neuroprotective, and wound-healing properties. Vegetables and fruits represent a great source of vitamin C, and its recommended ingestion is established as 90 mg/day for males and 75 mg/day for females [116].

The history of AA started with James Lind demonstrating that citrus fruit consumption had positive effects on scurvy prevention and treatment, and it was named as an antiscorbutic factor. Its structure was elucidated by Albert Szent-Györgyi in 1932 [117], who was awarded the Nobel Prize for Medicine in 1937.

The ascorbic acid biosynthesis has many routes. In mammals, it can be generated from D-glucose, which is converted to d-glucuronic acid, and later to l-gulonic acid by glucuronate reductase. This molecule is then turned to gulono-1,4-lactone by aldono-lactonase. Finally, ascorbic acid is converted through the action of gulono-1,4-lactone oxidase in gulono-1,4-lactone, producing 2-keto-gulono-*γ*-lactone, which spontaneously converts to l-ascorbic acid or vitamin C [118]. On the other hand, plants synthesize l-ascorbic acid from l-galactose, which comes from D-mannose. l-galactose is converted to l-Galactono-1,4-lactone via oxidation by NAD-dependent l-galactose dehydrogenase. l-ascorbic acid is finally formed via oxidation of l-Galactono-1,4-lactone by l-galactono-1,4-lactone dehydrogenase [119,120].

### 2.8. Curcumin

Curcumin ((1E,6E)-1,7-Bis(4-hydroxy-3-methoxyphenyl, CU) hepta-1,6-diene-3,5-dione, also known as diferuloylmethane) is a non-polar diarylheptanoid, natural, bright and yellow polyphenol from the *Curcuma* genus. It is the principal curcuminoid of turmeric (*Curcuma longa*), a member of the ginger family, Zingiberaceae, that is obtained mainly from its rhizome [121,122]. Curcuma has been used for years due to its beneficial properties for human health. Nowadays, it is an authorized food additive, labeled as E-100i (curcumin) and E-100ii (curcuma). It is used as an herbal supplement, cosmetics ingredient, food flavoring, and food coloring [123]. CU is present in at least two forms, the keto and enol tautomers, which are solid and liquid, respectively.

CU has anti-cancer [124,125], anti-arthritis, anti-inflammatory [126], and neuroprotective capacity, besides its antioxidant properties [127]. It aids in the management of oxidative and inflammatory conditions, metabolic syndrome, arthritis, anxiety, and hyperlipidemia. It may also help in the treatment of exercise-induced inflammation and muscle soreness, thus promoting recovery and subsequent performance in active people [128]. However, there are some factors that limit the bioactivity of curcumin, such as chemical instability, insolubility in water, absence of potent and selective activity, low bioavailability, limited tissue distribution, and extensive metabolism [129,130]. The bioavailability of curcumin depends on the delivery format, age, health condition, and human gender [131]. Some studies have shown its increased biodisponibility and its reduced degradation upon encapsulation [132,133].

The biosynthesis of curcumin remains still unclear. Peter J. Roughley and Donald A. Whiting proposed two possible mechanisms for its biosynthesis in 1973. One involves a chain extension reaction by cinnamic acid and 5-malonyl-CoA, that arylize into a curcuminoid. The second possible mechanism involves two cinnamate units coupled by malonyl-CoA. On the other hand, it is believed that plants start with p-coumaric acid instead of cinnamic acid [134].

A summary of the most important properties of all the selected antioxidants is provided in Table 1.

Given that G is a zero-gap semiconductor and an electroactive and transparent material, there are many possibilities for its application in biosensing applications. Its outstanding electrical conductivity and large specific surface have demonstrated the accurate, rapid, sensitive, and selective sensing ability of bioactive compounds. It presents enhanced sensitivity for a wide range of biomolecules when compared with other carbon materials such as CNTs, fullerenes, or amorphous carbon.

## 3. Graphene Functionalization Approaches

Pristine G sheets are hydrophobic in nature, so they cannot be dissolved in polar solvents. To make it soluble in common solvents, avoiding stacking between adyacent sheets, and hence to expand its range of applications, it can be functionalized via interaction with other molecules or polymers. Noncovalent functionalization by π-interactions is an attractive synthetic method because it offers the possibility of attaching functional groups to G without disturbing the electronic system. These include H-π, π–π, anion-π and cation-π interactions [139]. Complexes exhibiting the H−π interaction (190, 201–207) are of great interest since this type of interaction is also one of hydrogen bonds [140]. On the other hand, the π–π interaction is one of the most important driving forces for supramolecular self-assembly. When the countermolecule is a metal cation, a combination of electrostatic and induction energies dominates the cation−π interaction. Recently, anion−π interactions have been reported as a novel approach towards a new type of anion recognition, host architecture, and supramolecular self-assembly [141]. Overall, by controlling the relationships of several noncovalent interactions, novel organic nanostructures can be designed.

Typically, non-covalent strategies include solution mixing or in situ polymerization. The first requires both G and the functional molecule or polymer to be stably dispersed in a common solvent; it involves the dispersion of G in the appropriate solvent, the adsorption of the molecule (or polymer) to delaminated G sheets in solution, and the elimination of the solvent, resulting in sandwich-like nanocomposite [12]. On the other hand, in in-situ polymerization, G is first swollen within the liquid monomer, the initiator is subsequently added, and the polymerization begins either by heat or radiation. Nanocomposites with conductive polymers can also be produced via in situ electrochemical polymerization [142], which yields mechanically stable composite films that can be directly used as electrodes or energy devices.

The key aim of the covalent functionalization of pristine G with organic molecules or polymers is to improve its dispersibility in common organic solvents. Furthermore, groups such as chromophores provide novel properties that could be combined with G properties such as conductivity. In most cases, when organic molecules are covalently linked to the G surface, their aromaticity is perturbed, enabling the control of its electronic properties. The functionalization reactions include two general approaches: (a) formation of covalent linkages between free radicals or dienophiles and C=C bonds of G and (b) formation of covalent bonds between organic functional groups and the oxygenated groups of GO. On the one hand, free radicals and dienophiles can react with sp^2^ carbons of G via 1,3 dipolar cycloaddition, aryne, or nitrene addition [143]. On the other hand, organic functional groups can be anchored to the epoxy, carboxylic acid, ketone, or hydroxyl groups onto a GO surface. In particular, chromophores, including azobenzenes, porphyrins, and phthalocyanines, with outstanding optoelectronic properties, have been covalently attached to G nanoplatelets. [144]. Thus, GO can be functionalized with porphyrins through the formation of amide bonds between amine-functionalized porphyrins and carboxylic groups of GO. Besides, GO can be grafted to polymeric chains that have reactive species like hydroxyls and amines, in particular poly(ethylene glycol), polylysine, polyallylamine, and poly(vinyl alcohol). The polymer provides improved dispersibility in certain solvents and morphological characteristics, while G offers electrical and thermal conductivity and reinforcement of the stiffness and strength.

**Figure 2:**
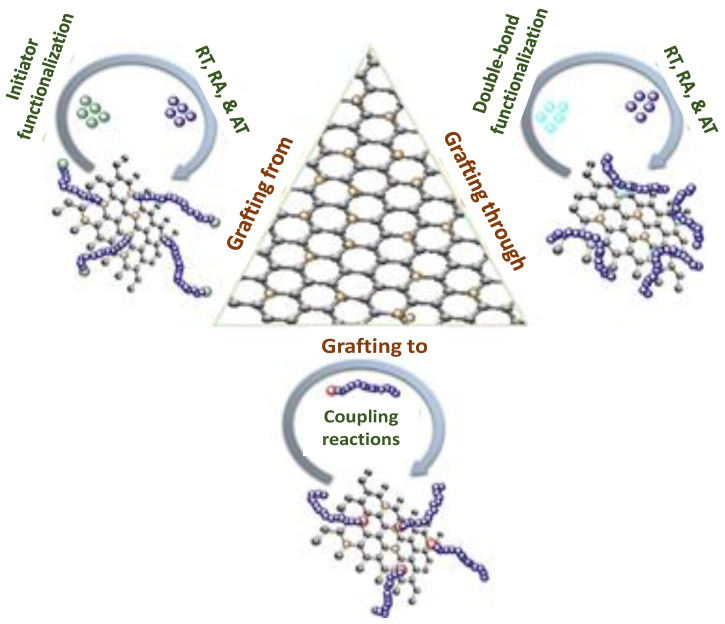
Representation of the anchoring of polymer chains onto graphene (G) via “grafting-to”, “grafting-from”, and “grafting-through” approaches. Adapted from Eskandari et al. [145].

Grafting of polymeric chains onto G nanosheets can be carried out via “grafting-to”, “grafting-from”, and “grafting-through” approaches (Figure 2) [145]. The first consists of synthetizing G and polymers individually and connecting them. Here, physical and chemical interactions play a role in modifying the G layers. In the “grafting-from” method, the polymer chains grow in situ from an initiator that has been previously anchored to the G surface. In the “grafting-through” approach, the polymerizable groups are anchored onto the G surface. Then, the polymerization begins in the solution that contains an initiator, monomers, and G. Polymerization of monomers takes place, and G is incorporated inside the polymer chains.

## 4. Graphene-Based Sensors for Bioactive Compounds

Due to its outstanding electrical, chemical, optical, and electrochemical properties, G has excellent potential for use as a transducer in optical sensors based on fluorescence, chemiluminescence, and colorimetric detection systems [35]. G derivatives, in particular GO and rGO, have valuable characteristics to be employed in these optical sensors. On the one hand, GO has tailorable luminescent properties. On the other hand, GO and rGO have been reported to be strong fluorescence quenchers via Föster resonance energy transfer (FRET) [146]. Based on this property, two types of G-based fluorescent sensors have been described: (1) signal-on ones, in which the fluorescence intensity rises with the addition of the analyte, hence the signal can be directly correlated with the analyte concentration reaching detection limits as low as ng/L. (2) signal off sensors, based on fluorescently labeled probes that adsorb onto GO in the presence of the analyte and quench its fluorescence, though have lower sensitivity than signal on sensors [30].

Furthermore, the application of G-based materials as sensors involves two approaches: one is based on G-biomolecule interactions via van der Waals, π-π stacking, cation−π, anion−π interactions, and electrostatic forces, leading to electrical variations in the pristine G. The other is based on the chemical functionalization to immobilize the molecular receptors onto the surface of GO, rGO, or GQDs [147].

From an electrochemical viewpoint, the potential of G-based electrodes is huge given that they preserve the properties of other carbon-based materials, including chemical inertness and good electrocatalytic activities for many redox reactions, and simultaneously they offer new properties like high surface area and ultrarapid charge mobility, which guarantee high sensitivity and quick response. Further, it presents an electrochemical potential window of ~2.5 V in 0.1 M PBS (pH 7.0) [148], which is better than that of graphite, glassy carbon, and even boron-doped diamond electrodes. Besides, the charge-transfer resistance on G is considerably lower than that of graphite or glassy carbon electrodes, indicating that the electronic structure of G is beneficial for electron transfer, making it suitable for the detection of biomolecules that have high oxidation or reduction potential. The key aspects of G electrochemistry are beyond the scope of this review and have been recently reviewed [149]. Furthermore, some former reviews have focused on the interactions of G, GO, and rGO-based biosensors with their analyte targets [150,151,152,153].

In the following section, we review the most recent advances in the development of optical and electrochemical sensors based on graphene and its derivatives for the detection of bioactive compounds. We discuss the processing and detection method, the linear range and limit of detection (LOD), as well as the advantages and improvement of properties due to the presence of graphene. Although the number of papers related to this type of sensor is still scarce, very promising results have already been obtained.

### 4.1. Melatonin

A lot of techniques to determine low concentrations of MLT in biological samples have been reported, including HPLC with electrochemical and fluorometric detection, gas chromatography-mass spectrometry, micellar electrokinetic chromatography, spectrofluorimetry, chemiluminescence, radioimmunoassay, and colorimetry [154,155,156,157,158]. However, some of these techniques have many drawbacks, such as the use of expensive instruments, regular maintenance, tissue destruction, tedious and complicated processes, and the use of organic solvents which are not biocompatible and generate pollution. Researchers in their investigations try to improve at least one of these disadvantages. Niu et al. [159] focused their work on the development of an optical sensor to detect MLT via a simple, cost-effective, and sensitive method. For such a purpose, they first synthesized GO via a modified Hummer´s method. Subsequently, it was dispersed in an aqueous medium and reacted with phenyl triethoxysilane (PTEOS) and tetramethoxysilane (TMOS) via a sol-gel method to yield a GO@SiO_2_ nanocomposite which was used as a sorbent in dispersive solid-phase extraction (dSPE). The detection of MLT was performed via HPLC combined with DAD, and a detection below 0.1 μg mL^−1^ was attained.

**Figure 3:**
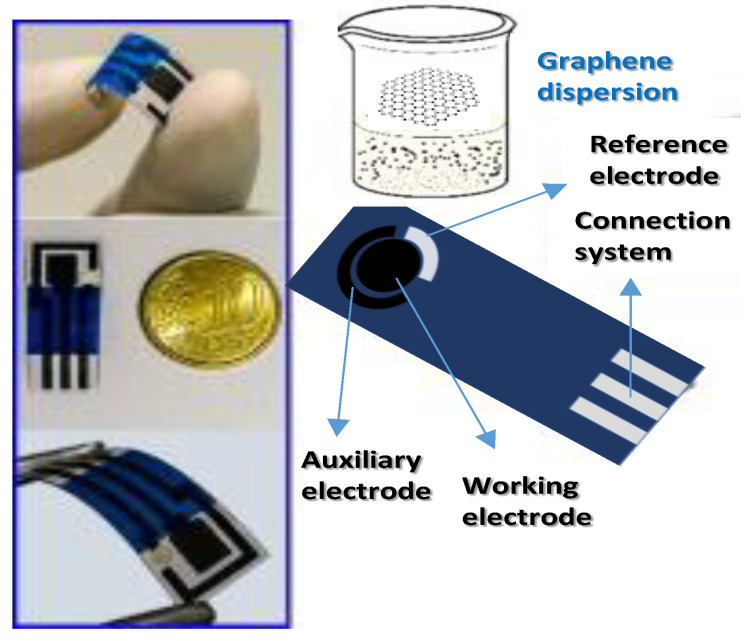
Graphene-coated carbon screen-printed electrode (G-CSPE) with the three-electrode system: a reference electrode, a working electrode, and an auxiliary or counter electrode.

However, most of the graphene-based sensors for MLT detection are based on electrochemical techniques, which are typically more sensitive, accurate, faster, miniaturizable, eco-friendly, and cheaper compared with optical ones. In this regard, Apetrei et al. [160] developed a novel sensor based on graphene-coated carbon screen-printed electrode (G-CSPE) prepared via sonication of G followed by drop-casting onto the electrode. These screen-printed electrodes (SPEs) consist of a single device with three different electrodes (Figure 3): (1) Working electrode, which response is sensitive to the analyte concentration. (2) The reference electrode, which potential is constant, and the working electrode potential is measured against it. Auxiliary or counter electrode, which completes the circuit of the cell, as it allows the passage of current. The voltammetric behavior of the SPEs (unmodified and modified with G) was studied in order to evaluate the electroactive surface area of the working electrode (using K_4_[Fe(CN)_6_] as benchmark redox system) and to quantify the rate constant obtained from cyclic voltammetric (CV) curves. The method was applied to the analysis of commercial pharmaceutical formulations.

The G-CSPE showed better performance, with a higher degree of reversibility, lower separation between the anodic and cathodic peaks, and the ratio between the current for the catodic and anodic peak (*I*_c_/*I*_a_) was close to 1. Good sensitivity in small samples was obtained with a detection limit of 0.87 µM and a response time of about 4 s.

A very similar approach was reported by Miccoli et al. [161] to detect MLT in food supplements using differential pulse voltammetry (DPV) with a G-CSPE. Graphene was suspended, and the electrodes were modified by drop-casting. Again, the electrode modified with CVD graphene led to better results than the unmodified one. Since the solution pH is crucial for the stability of melatonin molecules, studies were carried out at different pHs. They demonstrated how pH affects the relation between the signal peak and the amount of MLT. The detection limits for pHs at 6.4, 7.0, and 7.4 were 15, 30, and 60 µg/L, respectively, lower than those attained with the unmodified electrode.

Analogously, Gomez et al. [162] tested several carbon nanostructures to modify a CSPE and detect MLT and serotonin simultaneously in tablets and herb extract capsules. In particular, graphene oxide nanoribbons (GON) and graphene reduced nanoribbons (GRN) were synthesized from multi-walled carbon nanotubes (MWCNTs) via the longitudinal unzipping method, followed by chemical reduction with hydrazine, ultrasonication in aqueous media, and drop-casting onto the CSPE. The electrochemical behavior of the different electrodes was examined by DPV, reaching a LOD of 1.1 μM, with a low sample consumption (50 μL), good reproducibility, a response time of 120 s, and a recovery of 94–103%. The excellent performance obtained makes this approach promising not only in the pharmaceutical field but also in the determination of neurotransmitters in urine and other related samples.

Gupta et al. [163] prepared a sensor for the selective and sensitive determination of melatonin in human biological fluids based on the combination of rGO and a molecularly imprinted polymer (MIP). The rGO was synthesized from graphite powder via a modified Hummers´ method followed by hydrazine reduction. Then, the rGO was ultrasonicated in a mixture of distilled water and DMF (1:9) and dropped on the surface of a glassy carbon electrode (GCE). Subsequently, the MIP film was prepared by the electropolymerization on the surface of the modified electrode. The synergistic effect of graphene and the MIP enlarged the number of recognition sites, resulting in an improved MLT detection, with a linear range from 0.05 to 100 µM, and a LOD of 6 nM.

Another approach to improve the sensitivity of this type of sensor and enable the simultaneous determination of related molecules coexisting in biological systems is the combination of rGO with inorganic nanoparticles. In this regard, Bagheri et al. [164] developed an electrochemical sensor to detect MLT and dopamine, based on rGO decorated with Fe_3_O_4_ magnetic nanoparticles on a carbon paste electrode (CPE). The nanocomposite was prepared using a modified Hummers´ method followed by hydrazine reduction and then hydrothermal growth of the nanoparticles. Electrochemical studies revealed that the surface modification of the electrode considerably increased the oxidation peak currents, although it reduced the peak potentials of MLT and dopamine. The synergistic effect between the nanocomposite components enhanced the signal response, leading to a linear range of 0.02–5.80 µM and a LOD of 8.4 nM. Further, no significant effect on the recovery was found in the presence of interferences such as glucose, AA, pyridoxine, serotonin, or uric acid, among others.

**Figure 4:**
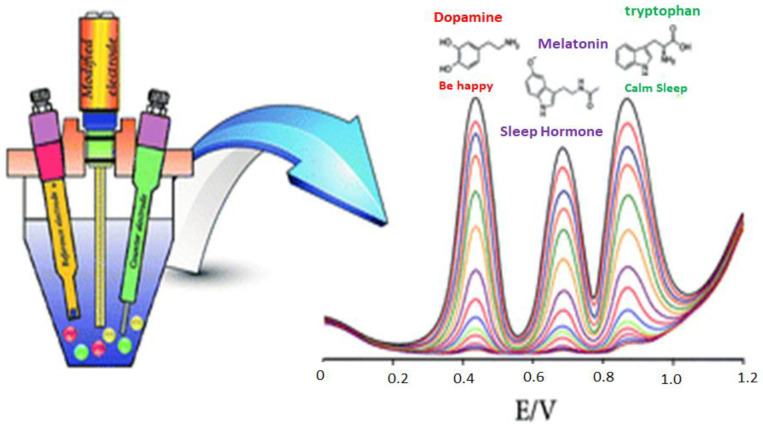
Differential pulse voltammetry (DPVs) for the determination of melatonin (MLT) at the *N*-reduced graphene oxide (rGO)/CuCo_2_O_4_/carbon paste electrode (CPE) in the presence of 2 mM dopamine and tryptophan. Taken from Tadayon et al. [166].

Other researchers such as Zeinali et al. [165] followed a similar method to detect tryptophan and melatonin at the time. They developed an electrochemical sensor with an ionic liquid carbon paste electrode modified with rGO and SnO_2_-Co_3_O_4_ nanoparticles. This SnO_2_-Co_3_O_4_@rGO/IL/CPE sensor worked linearly in the range of 0.02 to 6.00 µM, with a LOD of 4.1 nM, good selectivity, stability, and repeatability, besides its cost-effectiveness and simple fabrication. Furthermore, Tadayon et al. [166], based on the previous works, synthesized a sensor to detect dopamine, melatonin, and tryptophan. It was a nanocomposite based on nitrogen-doped reduced graphene oxide (N-rGO)/CuCo_2_O_4_ nanoparticles, deposited onto a CPE via a solvothermal method. The N-rGO improved the reactivity and electrocatalytic performance of the electrode, providing multiple binding sites, as well as enhanced biocompatibility and sensitivity. Both CV and DPV studies revealed that the potential separations between the three compounds were large enough to allow their simultaneous detection (Figure 4). Hence, it was employed for their analysis in human urine, serum, and pharmaceutical samples. Recovery values ranging from 97–104% were obtained, with a linear range of 0.01–3.0 µM and a detection limit of 4.9 µM for MLT. The cost-effectiveness and easy preparative method are valuable advantages of this sensor.

In the case of Liu et al. [167], a CuO−poly(l-lysine) (PLL)/graphene-sensing electrode for the detection in situ of MLT and pyridoxine (vitamin B_6_) was prepared via electrochemical deposition. CuO and PLL acted as linkers for the bioactive molecules, which were helped by a 3D graphene, grown via CVD, that amplifies the sensitivity. MLT was detected in a concentration range of 0.016−110 μM, with a LOD of 12 nM.

There are also a few articles that used MLT to reduce GO via an eco-friendly method [168,169]. Conventionally, GO is reduced by strong chemical agents, like hydrazine; however, they are not able to be produced at a large-scale due to their toxicity and harm to the environment. MLT has many advantages; the surface of MLT-reduced GO suspension presents more amount of nitrogen, attributed to the π–π adsorption, which triggers more stability; besides, when MLT is oxidized, it cannot be reduced back to its initial state, as lots of antioxidants do, protecting the rGO from oxidation. Ultimately, the efficiency obtained by MLT is comparable to the hydrazine one, although the deoxygenation process requires 600% more time.

### 4.2. Gallic Acid

Most of the reported studies focused on the determination of GA via electrochemical techniques (Table 2), which are simpler and provide higher sensitivity and selectivity. For instance, Al-Ansi et al. [170] synthesized a 3D nitrogen-doped porous graphene aerogel (NPGA) via one-step hydrothermal reduction by mixing graphene oxide (GO) with p-phenylenediamine (PPD) and ammonia solution and then followed by freeze-drying. The NPGA electrode provided a new way to determinate GA and showed improved analytical behavior compared to most of the electrodes reported in previous studies. It showed a large specific surface area, excellent electrical conductivity as well as high nitrogen content, and provided a linear range of detection from 2.5 to 1000 μM and a LOD of 67 nM.

**Figure 5:**
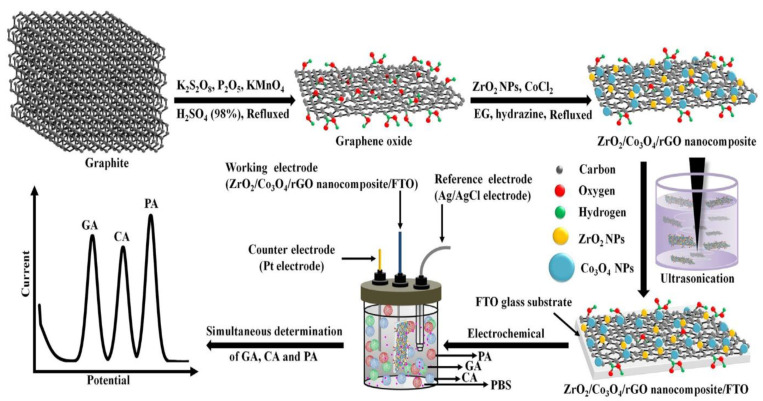
Schematic illustrations of the preparation of ZrO_2_/Co_3_O_4_/rGO nanocomposite and oxidation mechanisms of gallic acid (GA), caffeic acid (CA), and protochatechuic acid (PA). Taken from Puangjan et al. [172].

Chikere et al. [171] used amorphous ZrO_2_ nanoparticles decorated onto G to modify a carbon paste electrode. ZrO_2_ has a high surface area, good biocompatibility, good conductivity, and affinity for oxygen-containing groups, which resulted in an improved GA detection compared to the unmodified electrode. Thus, the mixing of zirconia and G produces an interaction that enhances the peak current of the oxidized GA. The electrode worked linearly in the range of 1 µM to 1 mM, with a LOD of 124 nM. Moreover, it was successfully applied for the determination of GA in red and white wines. A similar approach was used by Puangjan et al. [172], who synthesized an rGO/ZrO_2_/Co_3_O_4_ nanocomposite by a simple reflux method, as depicted in Figure 5. Thus, GO and ZrO_2_ nanoparticles were dispersed in ethylene glycol followed by the addition of CoCl_2_ and hydrazine hydrate and then refluxed at 80 °C. The hybrid nanocomposite exhibited a synergistic catalytic effect towards oxidation of GA, caffeic acid (CA), and protocatechuic acid (PA), with LODs of 1.56, 0.62, and 1.35 nM, respectively. The modified electrode was successfully applied for the simultaneous determination of the three species in fruit juice, rice and tea samples, showing rapid response and satisfactory recoveries.

**Figure 6:**
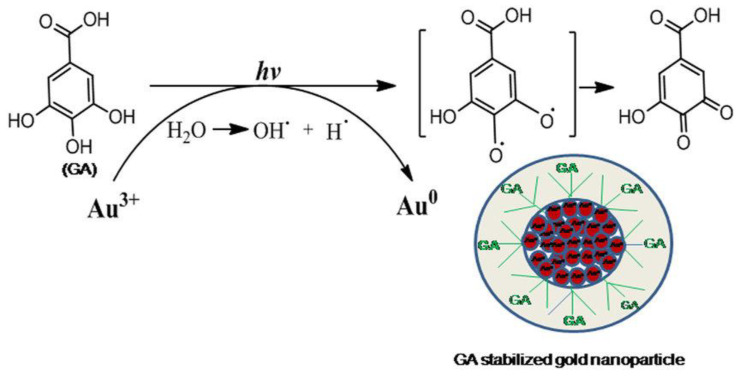
Diagram showing the formation of Au-nanoparticles (NPs) using GA under low-temperature sonication conditions. Adapted from Ganesh et al. [173].

On the other hand, Ganesh et al. [173] developed an MWCNT-rGO nanocomposite electrode for the sensitive detection of Au nanoparticles (NPs) capped with GA. The synthesis of this type of NPs capped with GA under low-temperature sonication conditions is depicted in Figure 6. The electrode was built by drop-casting an MWCNT-GO solution onto a GCE surface, followed by UV irradiation for GO reduction. DPV measurements were performed at different AuNPs-Ga concentrations, leading to a LOD of 2.57 pM, the lowest reported for this type of sensor. Using CV and electrochemical impedance spectroscopy (EIS), it was found that this modification of the electrode surface resulted in a 10-fold increase in the current response compared to unmodified electrodes. Thus, the capping of the nanoparticles allowed very sensitive and easy detection and prevented nanoparticle agglomeration. This green approach is interesting for the progress in nanotechnology, electronic, biomedical, and material science, in which metallic nanoparticles are increasingly used.

Electrochemical sensors based on hybrid materials comprising polymers and graphene derivatives have also been developed. Thus, Gao et al. [174] synthesized a nanocomposite incorporating chitosan (CS), fishbone-shaped Fe_2_O_3_ nanoparticles, and electrochemically reduced graphene oxide (ERGO) as the sensing matrix. The NPs were prepared via a solvothermal method; then, they were mixed with GO via ultrasonication, and the dispersion was drop cast onto a GCE, followed by electrochemical reduction. The electrochemical characterization experiments showed that the modified electrode had a large surface area, excellent electronic conductivity, and high stability. A good linear relationship between the oxidation peak currents in DPV and GA concentration was found in the 1–100 µM range, with a LOD of 0.15 µM.

**Figure 7:**
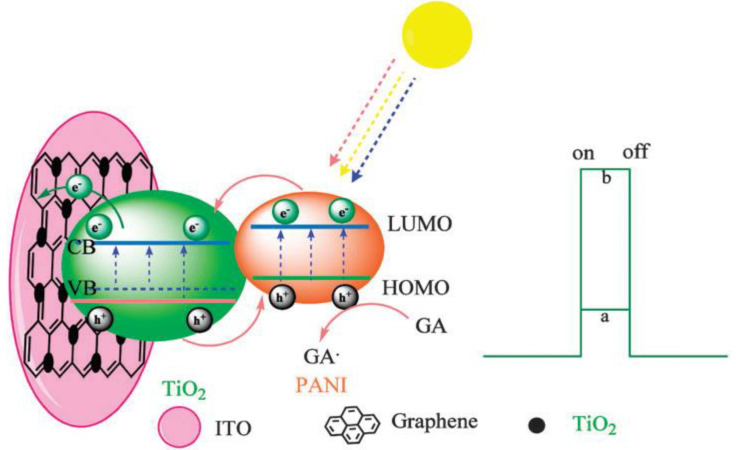
Schematic illustration of the photoelectrochemical process for GA oxidation at a polyaniline (PANI)–rGO–TiO_2_ modified electrode. Taken from Ma et al. [175].

Ma et al. [175] developed a photoelectrochemical sensor based on polyaniline (PANI)-rGO-TiO_2_ nanocomposite (Figure 7). PANI is an inexpensive and nontoxic conductive polymer with excellent stability, corrosion protection, and high mobility of charge carriers, hence highly suitable for photoelectronic materials. The nanocomposite was prepared via solvothermal synthesis of TiO_2_, followed by aniline polymerization, mixing, and ultrasonication. GA was detected in the linear range of 4.17 to 250 µM, with a LOD of 1.72 µM. This sensor showed a rapid response, high sensitivity, and excellent selectivity towards GA in the presence of other species such as AA, glutathione (GSH), and l-cysteine (CYs).

An optical sensor based on GQDs obtained by pyrolysis of citric acid was reported by Benítez-Martínez et al. [176]. GA was able to quench the GQDs fluorescence via π-π stacking and non-covalent interactions. The emission band (at 474 nm) underwent a green shift when GA from real samples is added. In addition, higher quenching was observed when the polarity of the solvent tested increased. The applicability of the method was evaluated on four different types of real olive oil samples, leading to a linear response over the concentration range 2–30 mg L^−1^ and a LOD of 0.3 mg/L^−1^. The proposed method was fast, very simple, sensitive, and reproducible.

On the other hand, a few papers have been reported on the use of GA for the development of sensors for the detection of other ions. For instance, Liu et al. [177] prepared a 3D-porous graphene-based hydrogel with good mechanical strength and large surface area, fabricated by self-assembly of GO sheets reduced and modified by GA through π-π interactions, to capture toxic Cr(III) ions generated by tannery wastewater. This GA-modified hydrogel is able to capture the Cr(III) by coordination complexation with its deprotonated carboxylic groups at pH around 4.0, with an average of nearly 97% of Cr(III) in 20 min. In addition, this functionalized structure is reusable due to its desorption with HCl at pH 2.0, releasing an average of 89% in 30 min. Both adsorption and desorption processes have improved compared to the unmodified hydrogel. Otherwise, some researchers employed the anticancer ability of this antioxidant. In this regard, Croitoru et al. [178] designed a multifunctional platform based on GO, synthesized by the traditional Hummers´ method, that acted as a nanocarrier where biologically active substances, such as GA could be loaded. Experimental results showed about 70% release in less than one day, 75% in four days, and 80% within 10 days. This novel nanocarrier could be useful to treat cancer or severe infections.

### 4.3. Tannic Acid

**Figure 8:**
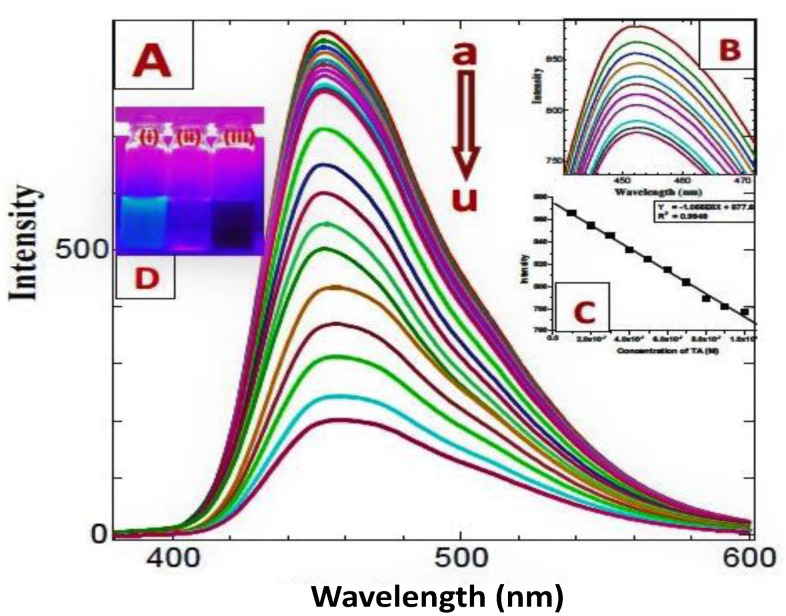
Fluorescence emission spectra of GQDs in the presence of different tannic acid (TA) concentrations, from 0.1 to 50 µM. The inset shows the linear plot of the intensity versus TA concentration. Taken from Sinduja et al. [179].

Although TA is a widely investigated molecule, very few papers regarding graphene-based sensors for TA determination have been published. Sinduja et al. [179] developed a colorimetric and a spectrofluorimetric sensor based on graphene quantum dots (GQDs) prepared via pyrolysis of citric acid. The mixture of GQDs and TA led to a new absorption band in the UV-Vis spectra due to the hydrogen bonding with the surface oxygen functional groups and π-π stacking interaction between aromatic groups of both compounds. On the other hand, the fluorescence intensity of GQDs linearly decreases while increasing TA concentration (Figure 8), from 0.1–1.0 μM with a LOD of 0.26 nM. Two quenching mechanisms have been proposed: (i) fluorescence resonance energy transfer (FRET), in which excited state electrons of GQDs return to the ground state via absorption of the energy emitted by the ground state electrons of TA transit to excited state resulting in a non-radiative process and (ii) simple charge transfer, in which the excited-state electron of GQDs meet TA, they transfer an electron to the Lowest Unoccupied Molecular Orbital (LUMO) of TA and then returns to ground state with a radiationless transition, which results in fluorescence quenching.

The electrochemical determination of TA using Zn-modified G electrodes has also been recently reported by Palisoc et al. [180]. The characterization via DPV led to a sensitive electrode, with a linear TA concentration range from 2 to 60 ppb and a LOD of 3.13 ppb.

On the other hand, several studies have been published on the use of TA as a stabilizer and reducing agent for G synthesis. For instance, Zhao et al. [181] used TA as a stabilizer for the preparation of high-quality graphene on a large scale through direct exfoliation of graphite via a green, high-efficiency, and low-cost method. Since TA acted as both dispersant and interfacial agent, G was uniformly dispersed and tightly integrated into polymer matrices for the development of high-performance and multifunctional nanocomposites. This environmentally friendly technique avoids the use of synthetic surfactants or organic solvents commonly employed for conventional G exfoliation in liquid media and prevents long reaction times. Analogously, Luo et al. [182] used TA as a reducing agent and stabilizer for GO synthesis and induced the self-assembly of rGO into a G hydrogel. The TA retained in the skeleton of 3D G also endowed the modified hydrogel with good antibacterial capability. Moreover, it showed excellent adsorption toward dyes, oils, and organic solvents; hence it is a promising candidate for efficient adsorbents in water purification. In another study, the same TA-modified hydrogel was used for the immobilization of Au-NPs. The obtained nanocomposite exhibited much higher catalytic activities than the bare NPs towards the reduction of methylene blue (MB). Overall, TA has been demonstrated to be an effective stabilizer for one-step exfoliation and noncovalent functionalization of graphene in aqueous media.

Another common application of TA is to aid in the development of several sensors. Lim et al. [183] prepared a humidity sensor based on a polyvinyl alcohol (PVA) nanocomposite filled with rGO coated with TA, which acted as a reducing and stabilizing agent and also increased the compatibility between rGO and the PVA matrix. The conductive property of rGO provides long-term stability, and the incorporation of rGO-TA into the PVA matrix enhanced mechanical strength. The PVA nanocomposite showed excellent humidity sensing properties over a wide relative humidity range. Similarly, Yoo et al. [184] synthesized an NH_3_ sensor based on TA-functionalized rGO, which demonstrated a high potential in gas sensing due to its high sensitivity, reversibility, and short response time.

### 4.4. Resveratrol

**Figure 9:**
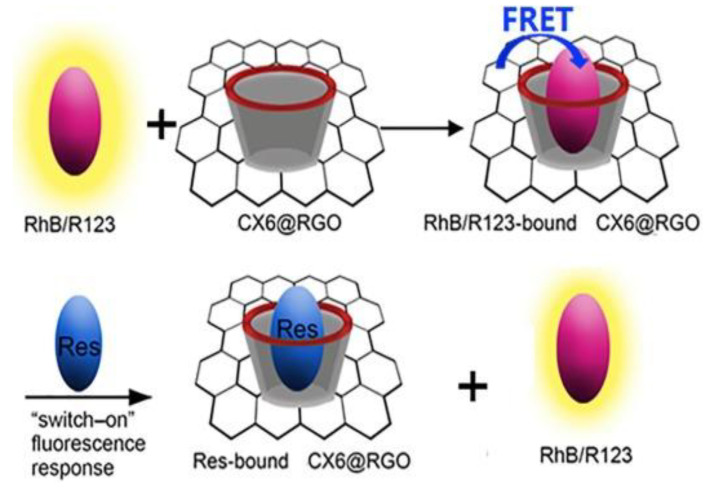
Displacement assay for resveratrol using calix(6)arene (CX6)-modified reduced graphene oxide (CX6@RGO) against a fluorescent dye. Taken form Li et al. [185].

The development of optical and electrochemical sensors for resveratrol (RES) determination has been the aim of a few studies. Thus, Li et al. [185] used the quenching property of GO to prepare a fluorescent FRET-based sensor via competitive supramolecular recognition between p-sulfonated calix(6)arene (CX6)-modified reduced graphene oxide (CX6@RGO) and a probe-resveratrol complex (Figure 9). The probe molecule, Rhodamine B (RhB) or rhodamine 123 (R123), had a strong fluorescence signal, and its fluorescence was quenched by CX6@RGO. However, if RES was added, the fluorescence reappeared proportionally to the amount of RES added. This was due to the new CX6@RGO-resveratrol generated, which inhibited the quenching. Fluorescence measurements were performed in the linear range of 2–40 µM, with a RES LOD of 0.47 µM.

Electrochemical methods have also been reported. Zhang et al. [186] used a direct laser-induced graphene (LIG) technique, which transformed the commercial Kapton/polyimide tape into 3D porous G. The prepared electrochemical sensor showed excellent repeatability, stability, reproducibility, and reliability, with an excellent linear response within the RES concentration range from 0.2 to 50 μM and a low LOD of 0.16 μM. Furthermore, the developed sensor was applied for the evaluation of RES levels in red wines and grape skins with outstanding results. On the other hand, Liu et al. [187] synthesized a sensor by one-step electrodeposition of rGO onto a GCE, which was compared with the bare GC electrode. The increased surface of rGO strongly enhanced the sensitivity of the sensor due to the π-π interaction between the rGO and RES. The response was linear in the range from 0.8 µM to 32 µM. Further, the electrode was stored at pH 2.0 and 4 °C for one month, and it retained a 95.6% of the original interaction. This approach of measuring RES using a G derivative is a cost-effective, eco-friendly, and effective technique.

On the other hand, RES has been used as a reducing agent for G derivatives. As mentioned earlier, GO is generally reduced by chemical methods; however, it results in limited solubility and an irreversible agglomeration of rGO due to the strong π–π stacking tendency between graphene layers. For that reason, surfactants are used. Another option to overcome this trouble is to employ a green reduction. In this regard, Gurunathan et al. [188] synthesized rGO from GO using RES [189]. Then, a RES-rGO complex was prepared to study its effect against ovarian cancer, and it exhibited much more cytotoxicity than just rGO, which is also known to decrease cell viability [190,191]. The incorporation of RES causes a significant toxicity increment, inducing cell death by promoting ROS generation.

RES and G nanocomposites have also been used for the treatment of other diseases. He et al. [192] used RES for its neuroprotective effect and GO, due to its properties and cost-effectiveness, to develop a structure able to recognize amyloid β (Aβ), closely implicated in Alzheimer’s disease. The Res@GO composite sensitively captured both Aβ monomers and fibers because RES is able to specifically bind with Aβ. Further, the fluorescence of RES was decreased with the GO addition via FRET. When Aβ was added, the fluorescence was restored due to RES removal. This approach based on the interaction between Aβ and the Res@GO complex was applied to detect an Alzheimer indicator via a quick and cost-effective method.

### 4.5. Oleuropein and Hydroxytyrosol

**Figure 10:**
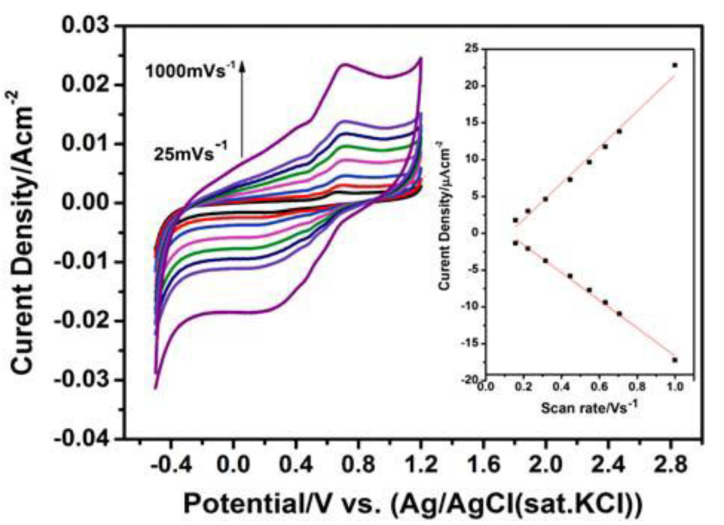
Cyclic voltammetric (CV) curves of 0.1 mM oleuropein (OL) on a TiO-rGO electrode at different scan rates from 25 to 1000 mV s^−1^. Taken from Yazar et al. [195].

Oleuropein (OL) is the ester of elenolic acid and HT and is one of the most significant components of the olive leaf extract. A few studies on the development of electrochemical sensors for OL and HT detection have been reported. Gomez et al. [193] developed a novel method for OL detection in complex plant matrices based on a Graphene Oxide Pencil Graphite Electrode (GOPGE). The electrochemical behavior of OL was examined using DPV, showing a signal enhancement of 5.3 times higher than the bare electrode. A calibration curve was performed between 0.10 to 37 μM, with a LOD of 30 nM. In another study, Kurtulbas et al. [194] developed an easy, accurate and sensitive detection method to determine OL using a TiOx-modified rGO glassy carbon electrode (TiOx-RGO@GCE). The rGO was prepared using AA as a reducing agent, and the nanocomposite via sol-gel method followed by drop-casting. CV and square wave voltammetry (SWV) experiments showed that the quasi-reversible reaction is the dominant mechanism on the electrode/electrolyte interface. A linear concentration range of 1–12 µM was obtained with a LOD of 18.7 nM. The same authors developed another TiO-rGO based electrode for OL detection, optimizing the synthesis conditions (Figure 10), and a linear concentration range of 5–30 nM was obtained with a LOD of 0.57 nM [195].

On the other hand, HT can be typically found in olive mill wastewater. For this reason, several studies have been reported on developing new methods for HT recovery. Sahin et al. [196] used GO synthesized by Hummer’s method as an adsorbent of HT. Adsorption of this bioactive compound from aqueous media onto GO was found to be >85% under optimum conditions. The pH of the adsorption medium was found to be a very important parameter affecting HT recovery. Increasing the pH from 3 to 9 increased the amount of adsorbed substance per GO from 0.55 to 89.46 mg. The same authors [197] also investigated a method to recover oleuropein and hydroxytyrosol from olive leaves and olive oil. For such purpose, a Zr-based metal-organic framework (UiO-66) and another based on graphene nanoplatelets (GNP/UiO-66). The use of GNP has been found to be more efficient than single-layer graphene, with excellent adsorption for organic pollutants because of its large, delocalized π-electron system. The particle size of UiO-66 was about 0.28 µm, which increased to 0.71 µm upon the addition of the GNP. Results showed that most of the hydroxytyrosol was removed from the solution, with an adsorption capacity of 142.07 mg per g UiO-66 nanoparticles at pH 10 in 180 min.

HT can also be used to reduce and stabilize GO. Baioun et al. [198] developed a green, low-cost, effective, and scalable method using an olive leaf aqueous extract rich in HT. It provided a high-efficiency removal of functional oxygen groups in the GO, generating and stabilizing rGO, which exhibited good solubility in aqueous solutions and some organic solvents.

### 4.6. Tocopherol

The determination of vitamin E has also been the aim of a few studies. Filik et al. [199] designed a Nafion (NF)/ERGO-modified GCE electrode. This nanocomposite provided excellent selectivity, sensitivity, stability, and reproducibility, and allowed the detection of TOH in the concentration range of 0.5 to 90 μM with a LOD of 0.06 μM. The electrode reaction of TOH is an irreversible process that takes place readily in the presence of water, free from interferences of other compounds such as AA.

**Figure 11:**
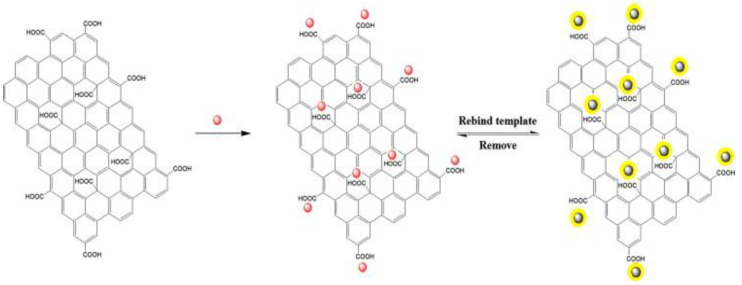
Representation of the synthesis of GO/QDs@molecular imprinted polymers (MIP) by a one-pot room temperature synthesis strategy with reverse microemulsion polymerization. Taken from Liu et al. [200].

MIPs have been combined with ionic liquids (ILs) to fabricate a GO/QDs nanocomposite sensor for the selective detection of traces of vitamin E in real samples. ILs are introduced on the GO surface by a one-pot room temperature synthesis strategy with reverse microemulsion polymerization (Figure 11) since they provide surface binding groups between GO and QDs, and also improve the fluorescence stability of GO due to their high thermal and chemical stability [200]. The fluorescence intensity of MIP was found to decrease with the increasing concentration of vitamin E in the range of 23–92 nM with a LOD of 3.5 nM and high precision. FRET is a possible mechanism for fluorescence quenching owing to no spectral overlap between the absorption spectrum of vitamin E and the emission spectrum of MIP.

### 4.7. Ascorbic Acid

Regarding AA determination, numerous methods have been reported in the literature; however, most of them present drawbacks such as high cost, operational complexity, laborious sample treatments, and high waste generation. Therefore, novel approaches are pursued. In particular, a few fluorescence sensors for AA determination have been reported. For instance, Liu et al. [201] developed a photoluminescent glycine (GLY)- functionalized GQDs by a simple and environmentally friendly pyrolysis method using ethylene glycol (EG) as a carbon source. The as-synthesized GLY-GQDs showed outstanding water solubility with a fluorescence quantum yield of 21.7%. The fluorescence of GLY-GQDs was quenched by Ce^4+^ via forming GLY-GQDs-Ce^4+^ non-luminescent complexes. Upon addition of AA, the fluorescence was restored due to the reduction of Ce4+ to Ce3 +. Based on this, a simple, fast, and inexpensive AA sensor was fabricated with a linear relationship in the range of 0.03–17.0 μM and a LOD of 25 nM without interference from other molecules such as uric acid dopamine, glutathione, and so on.

**Figure 12:**
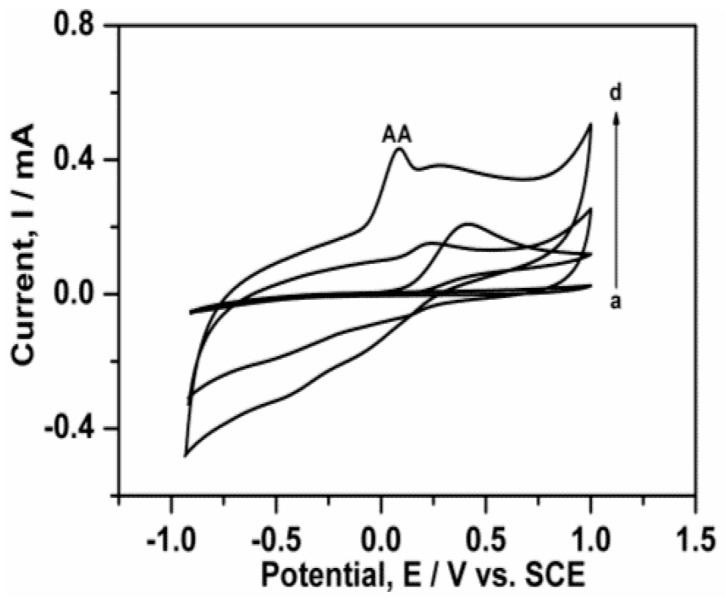
Cyclic voltammograms of (a) Bare graphite (b) Bare graphite with 5 mM ascorbic acid (AA) (c) NiO/G electrode in 0.1 M phosphate-buffered saline (PBS), pH 7.0 (d) NiO/G with 5 mM AA in 0.1 M buffer solution. Taken from Swamy et al. [203].

With regard to electrochemical methods, De Faria et al. [202] proposed a simple, sensitive, and precise approach using Flow injection analysis (FIA) with amperometric detection based on an rGO electrode prepared via simple dilution and drop-casting. The FIA system allowed a high analytical frequency, approximately 96 injections per hour, together with a linear concentration range of 65–253 μM and a LOD of 4.7 μM. Additionally, Swamy et al. [203] fabricated a sensor by decorating the surface of graphite electrode with NiO/G nanoparticles, which successfully separated the oxidation current signals of AA, dopamine, and tyrosine compared to a single, overlapped oxidative peak on a bare graphite electrode (Figure 12). The electrode has high selectivity and sensitivity (LOD of 50 µm) in addition to other factors like cost-effectiveness, convenience, and hassle-free electrochemical performance.

A very similar approach was applied by Kunpatee et al. [204], who used GQDs/IL-modified screen-printed carbon electrodes (SPCE) to determine AA, dopamine, and uric acid, which coexist in living systems. The GQDs/IL-SPCE exhibited excellent electrocatalytic activity for the oxidation of the three components in the mixture solution. Moreover, the anodic peak responses of the three analytes were well resolved into defined peaks. Under the optimal conditions, linear response for AA concentration was obtained in the range of 25–400 μM, with a LOD of 6.64 μM. The sensor exhibited high sensitivity, cost-effectiveness and was successfully applied for the simultaneous detection of these analytes in pharmaceutical products and biological samples. Analogously, Ji et al. [205] developed a smartphone-based integrated voltammetry system based on SPCE modified with rGO and electrochemically deposited. Experimental results corroborated that the system could be used to detect the electrochemical activity of these biomolecules with high sensitivity, linear and specific responses. Thus, AA was determined in the range of 20–375 μM, with a LOD of 1.04 μM.

**Figure 13:**
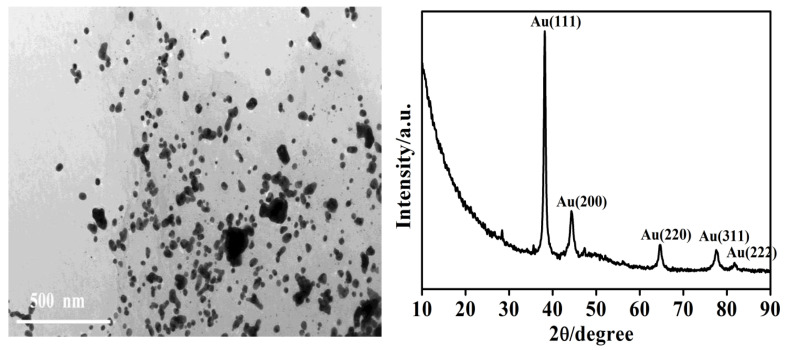
TEM images and X-ray diffraction (XRD) pattern of RGO/polydopamine(PDA)/Au nanohybrids. Taken from Shi et al. [207].

Fu et al. [206] also determined these three biomolecules using a G ink-coated glass prepared via simple water immersing followed by electrochemical reaction. CV studies revealed linear calibration curves in the range of 50–1000 μM, with a LOD of 17.8 μM. This study corroborated that the elimination of additives of the G ink upon film coating is a simple and cost-effective approach for sensor applications. Similarly, Shi et al. [207] detected them using rGO/polydopamine (PDA)/AuNPs nanocomposites prepared via reduction of GO nanosheets by PDA followed by mixing with the nanoparticles. The modified nanomaterials showed a big surface area, as revealed by TEM images (Figure 13), a high level of crystallinity according to X-Ray diffraction (XRD) analysis, exceptional biocompatibility and outstanding conductivity that promoted the electrocatalytic oxidation of the biocompounds, though the sensibility was not high. Thus, AA was only detected in the linear range of 4.93–9.60 mM, with an LOD of 1.64 mM.

**Figure 14:**
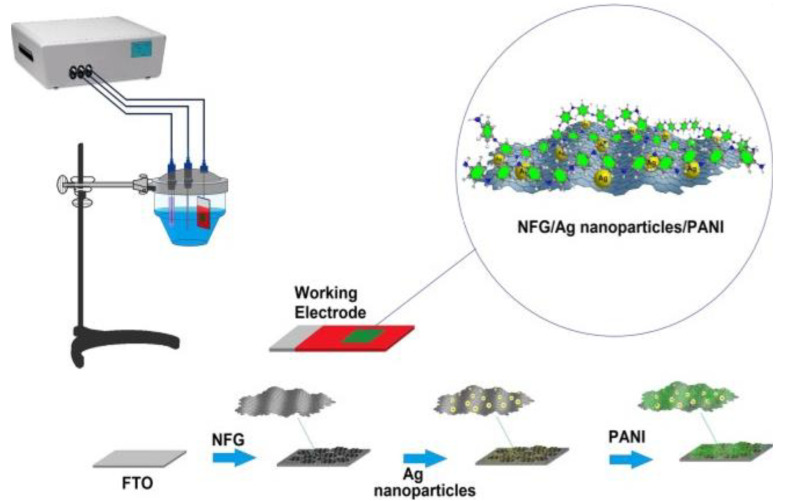
Representation of the synthesis of metal nanoparticles (NPs)-grafted N-doped functionalized graphene (NFG)/polyaniline (PANI) nanocomposites. Taken from *Salahandish* et al. [209].

Better sensitivity was attained by Li et al. [208], who developed a 3D nanocomposite based on MoS_2_ nanospheres, polyaniline (PANI), and rGO via a one-pot hydrothermal process. Thus, the peak currents obtained from DPV experiments varied linearly in the AA concentration range from 50 μM to 8.0 mM, with a LOD of 22.2 μM. The MoS_2/_PANI/rGO-based sensor exhibited high selectivity, reproducibility, good stability, and reliability for the trace determination of these three biocompounds. Another nanocomposite incorporating PANI was prepared by Salahandish et al. [209]. In particular, they synthesized a metal nanoparticle (NP)-grafted N-doped functionalized G (NFG)/PANI nanocomposite on a fluorine-doped tin oxide electrode (FTOE). The synthesis involved the coating of NFG on the FTOE substrate, chronoamperometry of metal NPs on the NFG-coated FTOE, and electropolymerization of PANI on AgNPs modified FTOE (Figure 14). A broad linear range was found between 10–11,460 µM, with a LOD of 8 µM. Results demonstrate that this nanocomposite is a suitable candidate for rapid, reproducible, and selective detection of AA in clinical samples.

Abraham et al. [210] developed a rGO/Pd-modified GCE to determine Epinephrine, AA, and uric acid, biomolecules that co-exist in the extracellular fluid of the central nervous system and serum. In this case, GO was synthesized via improved Hummer’s method and subsequently subjected to solar exfoliation by exposure to solar rays, which resulted in the formation of rGO. Then, it was suspended in methanol, drop cast on a GCE followed by electrodeposition of Pd. The metal incorporation resulted in improved electrochemical performance in terms of surface area and roughness. CV and DPV experiments were repeated at intervals of one, three, and six days giving reproducible results with an RSD of 2.4%. Thus, a linear range was attained from 300 to 1300 μM, with an LOD of 22 μM. Besides, the influence of pH on the oxidation behaviour was investigated, and it was found that the current increased to up a maximum at pH of 7. The sensor can be effectively used in real systems such as human blood serum and urine.

A more sensitive, inexpensive and reliable sensor was prepared by Kucukkolbasi et al. [211] based on a GO/CdTeQDs/GC electrode prepared via hydrothermal synthesis of the CdTeQDs followed by drop casting. CV and EIS experiments revealed that the modified electrode showed better performance than the bare GC one. The influence of pH buffer concentration, deposition potential, deposition time, and the presence of electroactive interferents on the response of the electrode was investigated. A linear response of the modified electrode was obtained over the concentration range of 32.3–500.0 µM with a LOD of 6.1 µM for AA.

The best sensitivity in the detection of AA has been reported by Chen et al. [212] using three-dimensional holey graphene (3D-HG), a 3D porous network prepared via wet-chemical etching with in-plane nanopores and a very large surface area that favors electrochemically active sites and increases electron-transfer rate. This sensor showed excellent properties for AA, uric acid, and nitrite detection using DPV, with a linear range of 3.2–0.2 µM and a LOD as low as 15 nM. Moreover, the applicability of the 3D-HG modified electrode was tested in real samples, showing very good accuracy and recovery. All the reported results corroborated that G-based materials are great candidates for the individual or simultaneous detection of dopamine, AA, uric acid, or nitrite, with high potential for future diagnosis. However, the real challenge still remains, that is, the development of an economical, reliable, and practical sensor with high sensitivity and selectivity.

On the other hand, several studies regarding the potential of AA as reducing agent for rGO environmentally friendly synthesis have been published. The first study that used AA for the non-toxic and scalable production of rGO was reported by Gao et al. [213], who employed this antioxidant to compete with the six traditional methods to prepare graphene from graphite oxide, in which toxic agents were used. l-AA was used as a reductant together with l-tryptophan, which acted as a stabilizer to avoid the agglomeration and precipitation of the resulting graphene sheets. AA reduces the reactive oxygen species in water, leading to a stable and unreactive process that does not cause cellular damage. Similarly, Fernández-Merino et al. [214] and Zhang et al. [215] developed novel green methods that lead to comparable reduction yields to hydrazine. Stable suspensions of AA-rGO can be prepared not only in water but also in common organic solvents; thus, this bioactive compound represents an ideal substitute for hydrazine in large-scale production. Another potential advantage of using AA as a reductant is that it is only composed of carbon, oxygen, and hydrogen, therefore minimizing the risk of introducing heteroatoms in the reduced products that were not present beforehand.

### 4.8. Curcumin

Electrochemical sensors have been used as worthy tools for the detection of CM due to their simplicity, accuracy, high sensitivity and selectivity, and reasonable price. Nevertheless, owing to the poor response of this compound, it is difficult to detect it directly at the surfaces of bare electrodes. In this regard, different modifiers have been used to solve this issue and to increase the sensitivity and selectivity of CM detection sensors. Rahimnejad et al. [216] developed a sensitive and accurate sensor based on rGO/CPE prepared via pulverization followed by drop-casting. Measurements via CV and DPV indicated a linear concentration range of 10–6000 µM, with a LOD of 3.183 µM. A similar approach was developed by Zhang et al. [217], who prepared a more sensitive voltammetric method for CM determination using an electrochemically reduced graphene oxide (ERGO)-modified GCE. The modified electrode showed much better electrocatalytic activity towards CM compared with bare GCE and GO/GCE electrodes. A linear voltammetric response was found from 0.2 μM to 60.0 μM, with a LOD of 0.1 μm. Similarly, Li et al. [218] prepared a more sensitive, selective, and accurate G/GCE sensor, which was characterized via CV, EIS, and linear sweep voltammetry (LSV). The currents measured by LSV displayed presented a good linear relationship with CM concentrations in the range of 5.0 × 10^−8^ to 3.0 × 10^−6^ μM, with a low detection limit of 0.03 μM.

**Figure 15:**
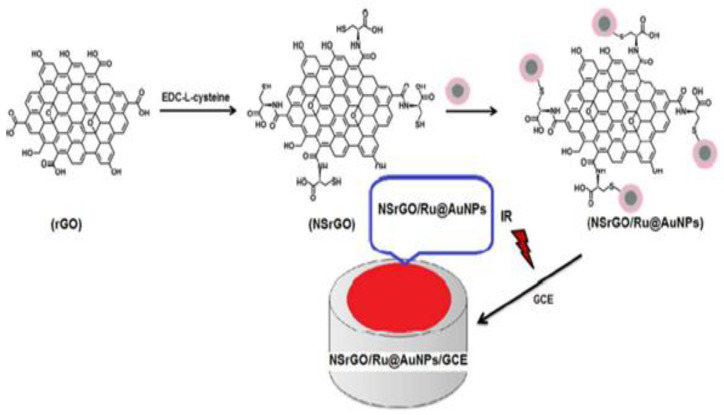
Schematic representation of the synthesis of l-cystein/rGO/Ru@AuNPs. Taken from Kotan et al. [220].

A comparative assessment of the potential of GO and rGO for electrochemical determination of CM was undertaken by Dey et al. [219]. GCE modified with these two nanomaterials was characterized using SEM, XRD, FTIR, and Raman techniques to understand their morphology and structure. rGO/GCE showed a lower limit detection of 0.9 pM and good signal quality. Further, the repeatability was checked for seven cycles, and interference studies corroborated the selectivity of the method. Even better sensibility was found by Kotan et al. [220], who synthesized L-cysteine functionalized rGO composites were prepared via activation of the carboxylic groups of rGO with ethylcarbodiimidehydrochloride (EDC) (Figure 15) followed by mixing with the Ru@AuNPs and ultrasonication and then drop-cast onto a GCE using an IR heat lamp. The electrochemical determination was studied using SWV with a linearity range of 0.001–0.1 nM and an unprecedented LOD of 0.2 pM.

On the other hand, other studies have been reported on the development of G/CM hybrids with antibacterial activity. Marković et al. [221] presented a G/CM nanomesh with antibacterial activity against Gram-positive bacteria like *Staphylococcus aureus*, with a minimum inhibitory concentration (MIC) of 1 mg mL^−1^. It was found that its cytotoxicity was concentration-dependent. At concentrations higher than 100 mg mL^−1^ some slight cytotoxic effects were observed. In this work, G was exfoliated from highly oriented pyrolytic graphite (HOPG) through an electrochemical exfoliation process with ammonium persulfate as an electrolyte, leading to CM/EHOPG nanomesh hybrids. Similarly, Bugli et al. [222] and Palmieri et al. [223] presented a method using Cm and Go to kill methicillin-resistant *Staphylococcus aureus* (MRSA).

Conversely, Yang et al. [224] developed a β-cyclodextrin (CD) functionalized GO nanocomposite, which displayed excellent antiviral activity and could load curcumin efficiently. Their aim was to find a new strategy to treat respiratory syncytial virus (RSV). Other researchers have employed CM to help in antitumoral treatments. Thus, Hatamie et al. [225] combined CM with rGO sheets, linked by π–π attachment, and studied its effects on human breast cancer cell lines and a normal cell line. Curcumin was utilized for simultaneous reduction of chemically exfoliated GO sheets and functionalization of the rGO ones. The interaction of the rGO sheets and cells resulted in apoptosis as well as a morphological transformation of the cells; thus, it could be used for nanotechnology-based bioapplications against cancer.

The most representative examples reported to date on optical and electrochemical sensors based on graphene and its derivatives for the detection of bioactive compounds are collected in Table 2.

**Table 2:** Characteristics of graphene-based sensors for the detection of bioactive compounds.

Bioactive Compound	Carbon Nanomaterial	Processing Method	Detection Method	Linear Range	LOD	Properties	Ref.
**Melatonin (MLT)**	GO@SiO_2_ nanocomposite	Modified Hummers´ + Sol-gel with PTEOS and TMOS	dsPE + HPLC with DAD	-	<0.1 µg/mL	Cost-effective, simple, selective and sensitive.	[159]
	G-CSPE	G Sonication + Drop-casting	CV and FPA	-	0.87 µM	Good sensitivity, reversibility, I_c_/I_a_ ≈ 1.	[160]
	CVD G-CSPE	G Suspension + Drop-casting	DPV	-	15 µg/L	Good sensitivity, reproducibility, versatility, better results than the electrode without G	[161]
	GON-CSPE GRN-CSPE	Longitudinal unzipping + hydrazine reduction + ultrasonication + drop-casting	CV and DPV	-	1.1 µM	Good reproducibility and response time, recovery of 94%–103%.	[162]
	rGO/MIP	Modified Hummers´ + hydrazine reduction + rGO Suspension + Drop-casting + electropolymerization.	CV and SWV	0.05–100 µM	6 nM	Stable and highly sensible.	[163]
	rGO/Fe_3_O_4_	Modified Hummers´ + hydrazine reduction + hydrothermal growth	SWV	0.02–5.80 µM	8.4 nM	Good selectivity, repeatability, reproducibility, and biocompatibility.	[164]
	rGO/SnO_2_-Co_3_O_4_ nanocomposite	Modified Hummers´ + SnO_2_ reduction + hydrothermal growth	CV and SWV	0.02–6.00 µM	4.1 nM	Good sensitivity, selectivity, stability, and repeatability; cost-effective and simple fabrication.	[165]
	N-rGO/CuCo_2_O_4_ nanocomposite	Modified Hummers´ + hydrazine reduction + solvothermal method	DPV and SWV	0.01–3.0 µM	4.9 nM	Enhanced selectivity, sensitivity, and biocompatibility.	[166]
	CVD G/CuO-PLL nanocomposite	CVD growth + electrochemical deposition	CV and SWV	0.016–110 μM	12 nM	Good sensitivity and biocompatibility.	[167]
	rGO	Modified Hummers´ + MLT reduction	CV	-	-	Simple, reproducible and biocompatible.	[168]
**Gallic acid (GA)**	NPGA	hydrothermal reduction of GO with PPD + freeze-drying	DPV and SWV	2.5–1000 μM	67 nM	Large specific surface area and excellent electrical conductivity.	[170]
	G/ZrO_2_	Hydrothermal growth + physical mixing	DPV and SWV	1µM–1 mM	124 nM	High surface area, good biocompatibility, and electrical conductivity.	[171]
	rGO/ZrO_2_/Co_3_O_4_	Modified Hummers´ + hydrazine reduction + ultrasonication + drop casting	CV and DPV	6.2–478 nM	1.56 nM	Good sensitivity, selectivity, reproducibility, and stability vs. interferences.	[172]
	MWCNT/rGO nanocomposite	Drop-casting + UV reduction	CV and EIS	29–329 pM	2.57 pM	Excellent sensibility, reproducibility, and long-term stability.	[173]
	CS/Fe_2_O_3_/ERGO nanocomposite	Solvothermal synthesis of Fe_2_O_3_ + ultrasonicaction + drop casting electrochemical reduction	DPV and EIS	1–100 µM	0.15 µM	Large surface area, excellent electronic conductivity, and high stability.	[174]
	PANI–rGO–TiO_2_	Solvothermal synthesis of TiO_2_ + aniline polymerization + mixing + ultrasonication	CV and PC	4.17–250 µM	1.72 µM	Rapid response, high sensitivity, and excellent selectivity.	[175]
	GQDs	Pyrolysis of citric acid	LLE + Fluorescence	5–40 mg/L	1.08 mg/L	Simple, sensitive, and reproducible. Fast response.	[176]
**Tannic acid (TA)**	GQDs	Pyrolysis of Citric Acid	UV-Vis and Fluorescence	0.1–1 µM	0.26 nM	Good selectivity and applicability.	[179]
	Zn-G	Electrolysis of graphite rods	DPV	2–60 ppb	3.13 ppb	Sustainable and cost-effective.	[180]
**Resveratrol (RSV or RES)**	CX6@RGO	Ultrasonication + mixing+ freeze drying.	UV-Vis and Fluorescence	2–40 µM	0.47 µM	Fast, simple, sensitive and selective.	[185]
	Porous G	Laser-induced conversion of Kapton/PI tape into 3D porous G	DPV	0.2–50 μM	0.16 μM	Excellent repeatability, stability, reproducibility, and reliability.	[186]
	rGO-GCE	Sonication + electrochemical deposition	CV and DPV	0.8–32 μM	0.2 μM	Long-term stability; low-cost, eco-friendly, and effective.	[187]
**Oleuropein (OL) and Hydroxytyrosol (HT)**	GOPGE	Sonication + drop casting	DPV	0.10–37 μM	30 nM	Good sensitivity and selectivity.	[193]
	TiOx-RGO@GCE	Hummer´s + reduction with AA + sol gel + drop casting	CV and SWV	1–12 μM	18.7 nM	Good sensitivity, simple and accurate.	[194]
	TiO-rGO	Hummer´s + reduction with AA + sol gel + drop casting	CV and SWV	5–30 μM	0.57 nM	Good sensitivity and selectivity.	[195]
	GONs	Ultrasonication + unzipping of MWCNTs + drop casting	CV, EIS, and DPV	-	-	Excellent performance and is fast.	[226]
**Tocopherol (TOH)**	NF/ERGO/GCE	ultrasonicaction + drop casting electrochemical reduction	DPV	0.5–90 μM	0.06 μM	Excellent selectivity, sensitivity, and reproducibility. Fast and cost-effective.	[199]
	ILs/MIP/GO/QDs	one-step polymerization	Fluorescence	23–92 nM	3.5 nM	Excellent photochemical stability and sensitivity.	[200]
**Ascorbic acid (AA)**	GLY-GQDs	pyrolysis with EG	Fluorescence	0.03–17.0 μM	25 nM	High sensitivity and selectivity.	[201]
	rGO	Dilution + drop casting	FIA with amperometric detection	65–253 μM	4.7 μM	Simple, sensitive and accurate, and precise.	[202]
	NiO/G	Coprecipitation synthesis of NiO + ultrasonication + drop casting	CV and DPV+ chronoamperometry	-	50 μM	Good selectivity and sensitivity, and cost-effective, easy to handle.	[203]
	GQDs/IL-SPCE	Pyrolysis of Citric Acid + drop casting	CV and EIS	25–400 μM	6.64 μM	High sensitivity and conductivity, good biocompatibility, cost-effective.	[204]
	rGO/AuNPs/SPE	G suspension + mixing electrochemical deposition	CV and DPV	20–375 μM	1.04 μM	High selectivity and sensitivity.	[205]
	Graphene ink coated glass	Water immersion + electrochemical reaction	CV	50–1000 μM	17.8 μM	Simple and cost-effective.	[206]
	rGO/PDA/AuNPs	GO reduction by PDA + mixing	CV + EIS	4.93–9.60 mM	1.64 mM	Good biocompatibility and conductivity.	[207]
	MoS_2_-PANI/rGO	one-pot hydrothermal synthesis + drop casting	CV and DPV	8 mM–50 μM	22.2 μM	High selectivity, good reproducibility, and stability.	[208]
	NFG/AgNPs/PANI	NFG coating on FTOE + electropolymerization of PANI	CV	10–11460 µM	8 µM	Good reproducibility and excellent selectivity.	[209]
	GCE/Pd/rGO	Sonication + electrodeposition	CV, DPV, and EIS	0.3–1.3 mM	22 µM	Fast response, good selectivity.	[210]
	GCE/GO/CdTeQDs	Hydrothermal synthesis + drop casting	CV + EIS	32.3–500 µM	6.1 µM	Inexpensive, reliable, and sensitive.	[211]
	3D-HG/GCE	Wet-chemical etching + drop casting	DPV	0.2 μM–3.2 mM	15 nM	High sensitivity and selectivity, excellent electrocatalytic activity.	[212]
**Curcumin**	rGO/CPE	Pulverization + drop casting	CV and DPV	10–6000 μM	3.18 μM	Good replicability catalytic activity, and storage stability.	[216]
	ERGO/GCE	Electrochemical reduction + drop casting	CV	0.2 μM–60 μM	0.1 μM	Good replicability and catalytic activity.	[217]
	G/GCE	Drop casting	CV, EIS	0.05–3.0 μM	0.03 μM	High selectivity and accuracy.	[218]
	rGO/GCE	Drop casting	CV, DPV	0.1 nM–10 nM	0.9 pM	Exceptional sensibility.	[219]
	NSrGO/Ru@ AuNPs	l-cysteine functionalization+ Ru@AuNPs grafting	SWV	0.001–0.1 nM	0.2 pM	Exceptional sensibility.	[220]

Phenyl triethoxysilane (PTEOS); Tetramethoxysilane (TMOS); Dispersive solid-phase extraction (dSPE); Graphene-coated carbon screen-printed electrode (G-CSPE); Cyclic voltammetry (CV); Fixed-potential amperometry (FPA); Current of anodic peak (*I*_a_); Current of cathodic peak (*I*_c_); Differential pulse voltammetry (DPV); Molecular imprinted polymer (MIP); Square wave voltammetry (SWV); Nitrogen doped reduced graphene oxide (N-rGO); poly(L-lysine) (PLL); Graphene oxide nanoribbons (GON); Graphene reduced nanoribbons (GRN); Diode Array Detection (DAD); Nitrogen-doped porous graphene aerogel (NPGA); p-phenylenediamine (PPD); Chitosan (CS); Electrochemically reduced graphene oxide (ERGO); Photocurrent measurements (PC); Graphene Quantum Dots (GQDs); p-sulfonated calix[6]arene (CX6); Nafion (NF); Polydopamine (PDA); Polyaniline (PANI); Nitrogen-doped functionalized graphene (NFG); Three dimensional holey graphene (3D-HG); fluorine-doped tin oxide electrode (FTOE); Flow injection analysis (FIA).

Overall, G-based nanomaterials with high specific surface area, excellent electrical conductivity, good stability, and unique mechanical properties have been found to have enormous potential for the determination of bioactive compounds. Compared to other carbon-based nanomaterials, they can provide more active sites, increase the electrochemical active surface area, improve the mass transport rate, and accelerate the electron transfer rate; hence, better and more reliable results have been obtained.

## 5. Outlook and Future Prospects

Over the last years, G and its derivatives have shown huge potential in the field of optical and electrochemical sensors. Owed to their exceptional electrical, chemical, and mechanical properties, G-based nanomaterials have already been used as sensors for detecting a wide range of analytes, including bioactive compounds, which are essential for human health owing to their multiple biological effects, including antioxidant activity. These sensors display outstanding performance compared to those based on conventional materials in terms of sensitivity, selectivity, response time, and long-term stability. In particular, it is possible to detect picomolar concentrations using electrochemical sensors based on MWCNT/rGO nanocomposites or l-cysteine functionalized-rGO/GCE. Currently, despite the benefits of optical detection, including high selectivity, immunity to electromagnetic interference, and a wide dynamic range, very few optical sensors have been designed for the detection of bioactive compounds. Covalent and non-covalent functionalization of G-based nanomaterials with organic or inorganic systems (i.e., polymers, nanoparticles) offer novel means for the development of the next generation of G-based sensors. However, despite most of the developed sensors represent an important proof-of-concept, the full potential of G-based sensors is far from being reached, and several issues have to be addressed prior to the commercial use of G-based nanocomposites:

(1) New manufacturing/fabrication routed to prepare high-quality G with tailored morphology, and electronic properties are required since the performance of G-based sensors is closely related to the nanomaterial characteristics, namely, purity, defect content, degree of functionalization, and structural morphology. From a practical perspective, novel technologies to produce sensors with reproducible and repeatable characteristics are needed. Thus, improvements in manufacturing to diminish variations among sensors and yield consistent performance, novel sensor designs, and operating modes are required. Likewise, more work on integrating signal conditioning and processing electronics to minimize performance variations, improve selectivity and operating lifetime would be valuable for practical applications.

(2) The functionalization and ultrasonication processes applied to G prior to and during the sensor fabrication may result in a strong reduction in electrical conductivity. Hence, G-based nanocomposites might present electrical properties that do not satisfy the requirements for sensor applications.

(3) Approaches that allow the large-scale synthesis of G at a relatively low cost are highly desirable. Despite significant efforts having been carried out in this direction, current methods are seriously restricted by their low efficiencies, which should be addressed for commercial applications. To the best of our knowledge, a reliable method able to supply the huge demand for pristine GO (or rGO) via an environmentally friendly approach, with a short-sonication time, viable washing steps, and high yield is still lacking.

(4) The actual specific surface area of G-based nanomaterials is considerably lower than the theoretical predictions due to the strong agglomeration tendency of the nanosheets via π-π stacking interactions and the mixing with organic molecules can make it worsen. While some achievements have been attained via the addition of stabilizers, these can have detrimental effects on sensor performance. In this regard, novel approaches to efficiently exfoliate the G sheets and in a green way are pursued.

(5) The toxicity of G-based nanomaterials is not clear yet. Despite considerable efforts in evaluating the potential impact of these nanomaterials on human health and the environment, results are frequently contradictory. They might cause cytotoxicity in humans, and this issue should be clarified. It is important to highlight that “graphene” is not a single nanomaterial but a group of materials, which accounts for the fact that their biological effects may vary depending on their intrinsic properties. A number of parameters, including processing method, lateral dimensions, level of functionalization, defect content, etc., can strongly influence the toxicity of these nanomaterials. Furthermore, their biodegradation mechanisms and extent remain unclear. The number of graphene layers, the average lateral dimension, and the atomic C/O ratio can play a key role in their biodegradability.

Overall, the development and widespread usage of G-based nanomaterials are largely hindered by the lack of techniques to provide simple, reproducible, and cost-effective sensors at a large scale. The research in this field is still in its infancy. More work is needed to ensure that the sensors are reliable, robust, and have non-toxic and easy manufacturability in a cost-competitive manner. Cost should be reduced in order to attain economic production of these nanomaterials with defined structures and properties at a large scale. In addition, a better understanding of G interactions with the bioactive compounds and the detection (or signal transduction) mechanisms are critical. Moreover, issues related to biodegradation and biocompatibility must be carefully considered, and challenges including device minimization, integration, durability, and lifetime should be addressed. Even so, it is expected that after comprehensive research in the field and continuous innovative efforts, sensors incorporating G-based nanomaterials could offer a new outlook for the detection of a variety of analytes, in particular bioactive compounds. This can be achieved with the collaborations between different disciplines and technologies.

## 6. Conclusions

In this review, the potential of G-based nanomaterials, namely GO, rGO, and GQDs, for sensing applications, in particular for bioactive compounds with antioxidant properties such as melatonin, gallic acid, tannic acid, resveratrol, hydroxytyrosol, tocopherol, ascorbic acid, and curcumin has been discussed in detail. The synthesis process, functionalization routes, and main properties have been summarized, with particular emphasis on their sensitivity and selectivity. The use of carbon nanomaterials has been demonstrated to be really useful to detect antioxidants through an easy, fast, and green technique, leading to better performance than sensors based on conventional materials. Moreover, the combination between these carbon nanostructures and the antioxidants has opened new properties and applications owed to synergistic effects. Besides, these antioxidants can be used to reduce GO via inexpensive and environmentally friendly methods. Finally, the future outlook for the development of G-based sensors for this type of biocompounds has been outlined. The extensive research progress in nanotechnology for graphene nanomaterials will enable the development of highly sensitive, specific, and green nanosensors at an affordable cost.

## Figures and Tables

**Table 1:** Biological and physical properties of the antioxidants, their nutrient sources, and medical uses.

Antioxidant	Biological Properties	Physical Properties	Nutrient Sources	Functions and Medical Uses	Ref.
Melatonin	Immunomodulatory Thermoregulatory Anti-aging Anticancer Photo-and radioprotective Cardioprotective Antiarrhythmic agent	Off-white powder M_w_ = 232.28 g/mol d_25 °C_ = 1.175 g/cm^3^ T_m_ = 117 °C b.p. = 512.8 °C S_20 °C_ = 2.0 g/L	Coffee, Tea Red wine, Beer Banana Tomatoes Rice, Wheat Corn, Oat	Control of hypertension, obesity, and metabolic syndrome Modulation of inflammatory markers Modulation of oxidative stress Sleep disorders/Insomnia treatment Parkinson and Alzheimer diseases	[52]
Gallic Acid	Antimicrobial Anticancer Antifungal Antiviral Astringent Antiallergic Antiinflammatory Antimelanogenic Antiulcerogenic	Crystalline white powder M_w_ = 170.12 g/mol d_25 °C_ = 1.694 g/cm^3^ T_m_ = 260 °C b.p. = 501 °C T_d_ = 237.5 °C pK_a_ = 4.40 S_20 °C_ = 11.9 g/L	Blueberries Apples Flax seeds Tea, Coffee Walnuts Watercress Grapes, Wine Grenade	Control of periodontal disease Cell death in e human cancer cells Regulation of the genes involved in the cell cycle Prevention of degenerative diseases Prevention of cardiovascular diseases Inhibitor of diabetes dysfunction Inflammation suppressor	[57]
Tannic Acid	Astringent Chemotherapy drug enhancer Antiallergic Anticarcinogenic, Antimutagenic Antiinflammatory	Light yellow amorphous powder M_w_ = 1701.19 g/mol d_25 °C_ = 2.12 g/cm^3^ T_m_ = 218 °C T_d_ = 199 °C pK_a_ = 10 b.p. = 218 ° C S_20 °C_ = 250 g/L	Red wine Coffee, Tea Guava Spinach Black raisins Oaks Nuts Persimmon	Inhibitor of NO_2_ production Clarifying agent in wine and beer Flavoring agent in foods Treatment of diarrhea Topical to dress skin burns Treatment of rectal disorders.	[135]
Resveratrol	Anticancer Antiallergic Antiinflammatory Cardioprotective Inmunostimulatory Antimicrobial Antiplatelet agent Antifungal	White to yellow powder M_w_ = 228.25 g/mol d_25 °C_ = 1.40 g/cm^3^ T_m_ = 263 °C T_d_ = 222 °C b.p. = 449 ° C S_20 °C_ = 0.03 g/L	Peanuts Pistachios Grapes, Wine Blueberries, Cranberries Cocoa Chocolate	Natural reducing agent Prevention of cardiovascular disease Parkinson and Alzheimer diseases Regulation of triglycerides Inhibitor of platelet aggregationInhibitor of DNA duplication in cancer cells	[21,25,136]
Hydroxytyrosol	Immunostimulant Antimicrobial Antifungal Cardioprotective Anticancer Antiinflammatory Hepatoprotective Neuroprotective	White powder M_w_ = 154.16 g/mol d_25 °C_ = 1.30 g/cm^3^ T_m_ = 55 °C T_d_ = 361 °C b.p. = 355 ° C S_20 °C_ = 50 g/L	Olive leaves Olive oil Wine	Prevention of sexual dysfunctions Prevention of atherosclerosis Inhibitor of platelet aggregation Inhibitor of human LDL oxidation Stabilizer and antioxidant in foods	[88,137]
Tocopherol (Vitamin E)	Antiageing Anticancer Cardioprotective Antiinflammatory	Yellow- brown liquid M_w_ = 430.71 g/mol d_25 °C_ = 0.95 g/cm^3^ T_m_ = 2 °C b.p. = 220 ° C S_20 °C_ = 0 g/L	Nuts Avocado Salmon Mango Tomato Spinach Seed oils	Prevention of macular degeneration Prevention of Alzheimer’s disease Prevention of cardiovascular diseases Inhibitor of platelet aggregation Moisturizers/creams	[138]
Ascorbic acid (Vitamin C)	Antiageing Wound healing Anticancer Immunostimulant Neuroprotective	white powder M_w_ = 176.12 g/mol d_25 °C_ = 1.65 g/cm^3^ T_m_ = 190 °C b.p. = 553 °C S_20 °C_ = 330 g/L)	Guava Pepper Citrus Broccoli Grape Cauliflower Strawberry Mango	Prevention of Hepatitis Promotion of collagen synthesis Prevention of Alzheimer’s disease Reparation and maintenance of skin, blood vessels, scars, tendons, ligaments, etc. Cofactor in many enzymes Natural reducing agent Cell division and growth regulation	[118]
Curcumin	Anticancer Antiarthritis Antiinflammatory Neuroprotective	yellow crystalline solid (keto-) or liquid (enol-) M_w_ = 368.38 g/mol d_25 °C_ = 1.3 g/cm^3^ T_m_ = 183 °C b.p. = 591 °C S_20 °C_ = 0 g/L	Curcuma Curry Tea	Colitis and stomach ulcer protection Inhibitor of diabetes dysfunction Inflammation suppressor Treatment for viruses and pulmonary fibrosis Prevention of cancers Cosmetics ingredient Food flavoring and coloring	[128]

S_20 °C_ = solubility in water at 20 °C; M_w_ = Molecular weight; d_25 °C_ = density at 25 °C; T_m_ = melting temperature; b.p. = boiling point; T_d_ = decomposition temperature.
